# Nanoporous Frameworks with High Porosity and Unexpected Rigid Framework Topologies Based on Odd‐Numbered Ring‐Expanded Linkers

**DOI:** 10.1002/smsc.202300158

**Published:** 2023-11-20

**Authors:** Bodo Felsner, Karuppasamy Gopalsamy, Volodymyr Bon, Irena Senkovska, Guillaume Maurin, Stefan Kaskel

**Affiliations:** ^1^ Technische Universität Dresden Inorganic Chemistry I Bergstraße 66 01069 Dresden Germany; ^2^ ICGM University of Montpellier, CNRS, ENSCM F-34293 Montpellier France

**Keywords:** bff, catenations, dihydro azepine rings, high porosities, metal–organic frameworks (MOFs), new topologies

## Abstract

Two tetracarboxylic linkers containing seven‐membered rings are synthesized and utilized in metal–organic framework (MOF) syntheses, leading to the two new compounds DUT‐184 and DUT‐193. The introduced odd angles between carboxylates in a new dihydro‐azepine‐containing linker lead to exotic Zn‐based MOFs: a 3D structure based on two catenated sets of 2D nets in DUT‐184 and a cubic structure with so far unknown topology in the DUT‐193 framework. The mesoporous DUT‐193 shows high porosity upon adsorption of N_2_, Ar, CH_4_, and CO_2_ at their standard boiling points. Grand canonical Monte Carlo simulations show good agreement between experimental and theoretical results and shine light on the microscopic adsorption mechanism at low pressure.

## Graphical abstract

Seven‐membered rings are introduced into new linkers of metal–organic frameworks, leading to beautiful new zinc‐based structures with interesting topologies including the highly porous and rigid new topology **bff**.

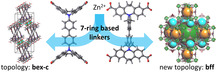

## 1 Introduction

Metal–organic frameworks (MOFs) are crystalline materials with ultrahigh specific surface area (SA)^[^ ^1^ ^]^ representing designed adsorbents with outstanding performance in gas storage,^[^ ^2^ ^]^ separation,^[^ ^3^ ^]^ and sensing,^[^ ^4^ ^]^ as well as drug delivery^[^ ^5^ ^]^ and catalysis.^[^ ^6^ ^]^ The design principles of MOFs, such as the isoreticular approach,^[6a]^ supramolecular building approach,^[^ ^7^ ^]^ and post‐synthetic modification^[^ ^8^ ^]^ including linker exchange^[^ ^9^ ^]^ and metal exchange,^[^ ^10^ ^]^ offer the potential to introduce desired properties and functionality as well as the opportunity to gain control over the targeted framework. In particular, the principles of isoreticular chemistry have been tremendously successful.^[^ ^11^ ^]^ In that case, frameworks of the same topology are created using linkers differing in size but with a common geometry/connectivity. Most prominent examples of isoreticular series are the isoreticular metal–organic framework (IRMOF) series based on MOF‐5 (**pcu** topology)[^11^, ^12^] or MOF‐74/CPO‐27 (**etb** topology),^[11b]^ the series of DUT‐49 analogs (**fcu‐a**),^[^ ^13^ ^]^ the NOTT‐100–NOTT‐109 series (**nbo**),^[^ ^14^ ^]^ as well as the porous coordination network (PCN) series (**rht**).^[^ ^15^ ^]^

Since only the linker is modified in isoreticular MOFs, the properties, such as framework topology, crystal structure, mechanical stability, and porosity of the resulting frameworks, are relatively straightforward to predict based on the parent MOF. However, finding novel framework properties relies on the design of new topologies and pore systems. One way to do that was summarized by Guillerm and Maspoch and termed “geometry mismatch”.^[^ ^16^ ^]^ For that strategy, known metal clusters are combined with atypical linkers that are twisted or bent in the case of ditopic linkers or just contain odd or unequal angles between coordinating groups in the case of polytopic linkers.^[^ ^16^ ^]^

**Figure 1:**
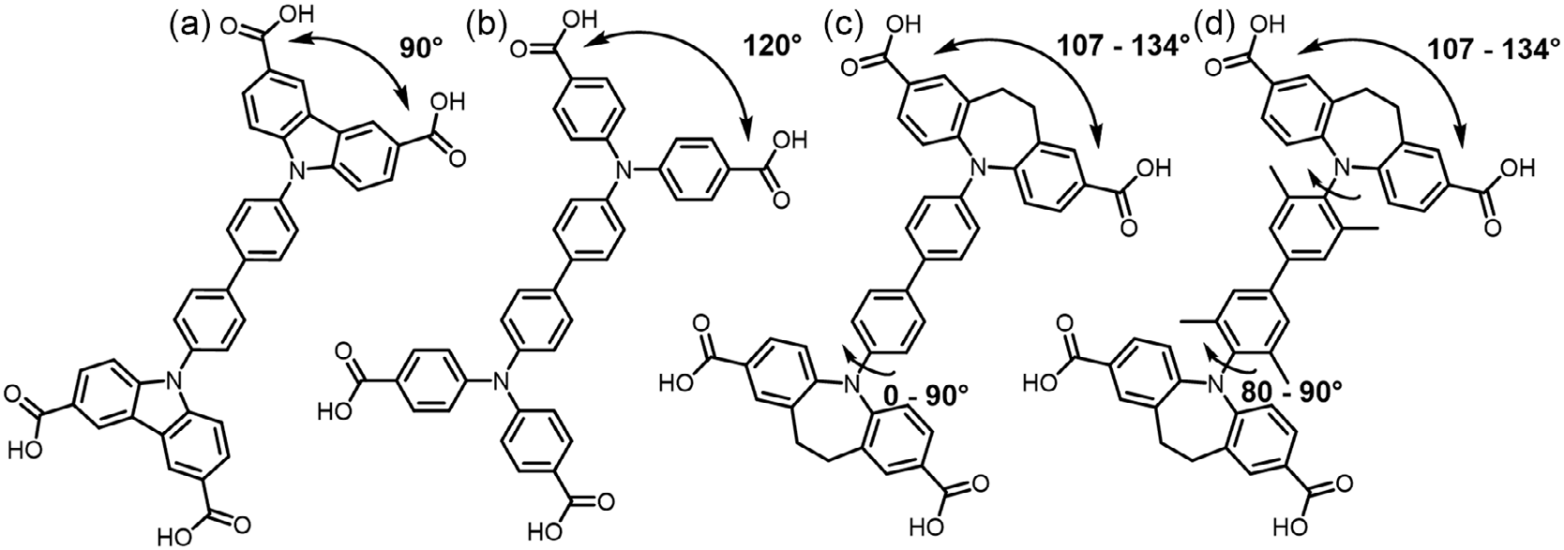
a–d) The structure of: a) the H_4_BBCDC linker of DUT‐49, b) the H_4_BenzTB linker of DUT‐10, DUT‐11, DUT‐12, and DUT‐13, and c,d) the two new linkers: c) 5,5′‐([1,1′‐biphenyl]‐4,4′‐diyl)bis(10,11‐dihydro‐5*H*‐dibenzo[*b*,*f*]azepine‐2,8‐dicarboxylic acid) (H_4_BBDDADC) and d) 5,5′‐(3,3′,5,5′‐tetramethyl‐[1,1′‐biphenyl]‐4,4′‐diyl)bis(10,11‐dihydro‐5*H*‐dibenzo[*b*,*f*]azepine‐2,8‐dicarboxylic acid) (H_4_TBBDDADC).

In recent years, we systematically used a number of tetratopic carboxylate linkers for the design of MOFs with ultrahigh porosity and flexibility, namely a series of linkers based on differently bridged bis(9*H*‐carbazole‐3,6‐dicarboxylates) (CDC^2−^) resulting in MOFs of the DUT‐49 series^[^ ^13^, ^17^ ^]^ and the *N,N,N′,N′*‐benzidine tetrabenzoate (BenzTB^4−^) linker in DUT‐10–DUR‐13 and DUT–25).^[^ ^18^ ^]^ Except for DUT‐13 and DUT‐25, which are based on the Zn_4_O^6+^ metal cluster, the remainder of the mentioned MOFs is based on paddle wheel dimers containing different metals. The CDC^2−^‐based linkers (i.e., 9,9′‐[1,1′‐biphenyl]‐4,4′‐diyl)bis(9*H*‐carbazole‐3,6‐dicarboxylate [H_4_BBCDC] in DUT‐49) show a fixed angle of around 90° between their carboxylate groups, while the more flexible BenzTB linker leads to angles around 120° (**Figure** **1**).

The introduction of a seven‐membered dihydro azepine ring to connect the outer phenyl rings of the linker should lead to higher and atypical angles between the carboxylate groups and therefore introduce a certain degree of geometry mismatch with both paddle wheel and M_4_O^6+^ clusters expected to result in new topologies.

The underlying topology of each structure is determined by the connectivity of its secondary building units (SBUs) and is usually described by its three‐letter reticular chemistry structure resource (RCSR) symbol.^[^ ^19^ ^]^ As an example, an M_2_ paddle wheel SBU with four‐connected carboxylate groups can be simplified as a four‐connected node in the net or a square unit in the augmented net, while most tetratopic carboxylate linkers also can be considered as four‐connected nodes, but depending on their geometry are described as tetrahedra or squares in the augmented net.^[^ ^20^ ^]^ Since the simplification of polytopic linkers is ambiguous, Proserpio et al. summarized different approaches for simplifications authors usually use. They are 1) the tertiary building unit (TBU) approach, where a TBU such as a metal–organic polyhedron is considered as a single node that is connected by other nodes or links; 2) the “single node” approach, where each metal cluster and each polytopic linker resembles a single node each; and 3) the “all node” approach, where each branching point in the structure is assigned to a separate node, oftentimes dividing polytopic linkers into smaller subunits.^[^ ^12^ ^]^

The application of these three methods to DUT‐49 leads to the different topologies **fcu**, **nbo**, and **tfb**, respectively.^[^ ^12^, ^21^ ^]^ All of these are reasonable simplifications but contain different information: **fcu** is well suited for the explanation of the flexibility mechanism in DUT‐49, as mainly the linkers are distorted in the course of framework deformation.^[^ ^13^, ^17^, ^22^ ^]^ At the same time, the **tfb** topology is required to identify isoreticular structures since only the most detailed simplification can do so.

In this work, we aim to point out the influence of a geometry mismatch in tetratopic linkers by the introduction of seven‐membered rings, which, to the best of our knowledge, were never employed in MOF chemistry up to now. Additionally, we introduce sterically demanding methyl groups into the biphenyl moiety of the linker to create a twist of the outer dicarboxylate units to further increase a mismatching geometry. Such isoreticular approach was utilized in a similar fashion for the design of DUT‐149, a compound isoreticular to DUT‐49.^[17f]^

In the following, we report the synthesis and characterization of two new linkers 5,5′‐([1,1′‐biphenyl]‐4,4′‐diyl)bis(10,11‐dihydro‐5*H*‐dibenzo[*b*,*f*]azepine‐2,8‐dicarboxylic acid) (H_4_BBDDADC, Figure 1c) and 5,5′‐(3,3′,5,5′‐tetramethyl‐[1,1′‐biphenyl]‐4,4′‐diyl)bis(10,11‐dihydro‐5*H*‐dibenzo[*b*,*f*]azepine‐2,8‐dicarboxylic acid) (H_4_TBBDDADC, Figure 1d) and their resulting new MOFs [Zn_2_(BBDDADC)(NMP)_2_]_*n*_ (DUT‐184, NMP: *N*‐methyl‐2‐pyrrolidone) and [Zn_4_O(TBBDDADC)_3/2_]_*n*_ (DUT‐193). The introduced atypical seven‐membered ring motif in DUT‐184 leads to a barely porous 3D network consisting of catenated 2D layers. In the case of DUT‐193, the introduction of additional methyl groups in the biphenyl bridge leads to a totally different highly porous network with so far unknown network topology by “all node” simplification. The porosity of new compounds was proved by physisorption of N_2_ (77 K) and Ar (87 K). Additionally, DUT‐193 was investigated upon methane (91 and 111 K) and CO_2_ (195 K) adsorption. Both physisorption and computational analysis shine light on the adsorption mechanism in the trimodal mesopore system of DUT‐193.

## 2 Results and Discussion

### 2.1 Syntheses of the Linkers

The syntheses of the linkers were performed following the scheme shown in Figure S1, Supporting Information. Starting from 10,11‐dihydro‐5*H*‐dibenzo[*b*,*f*]azepine, the substituted precursor dibutyl 10,11‐dihydro‐5*H*‐dibenzo[*b*,*f*]azepine‐2,8‐dicarboxylate (^*n*^Bu_2_DDADC) is synthesized over a four‐step route. Subsequently, the corresponding biphenyl bridges were coupled twice with the substituted azepine using a Buchwald–Hartwig coupling reaction with the specialized Pd‐RuPhos‐G4 catalyst that was reported to be highly active for azepine derivatives.^[^ ^23^ ^]^ The tetracarboxylic acids H_4_BBDDADC and H_4_TBBDDADC were obtained after hydrolysis of the corresponding esters (for more details, see Experimental Section).

### 2.2 DUT‐184 Structure and Porosity

**Figure 2:**
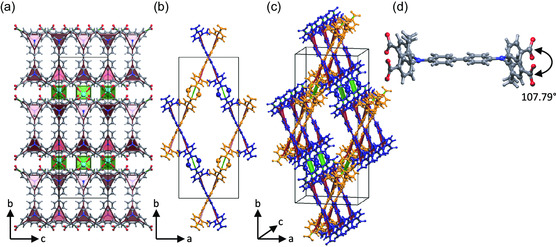
Structure and underlying augmented net of DUT‐184 (NMP molecules are omitted): a) along the *a*‐axis; b) along the *c*‐axis; c) 3D perspective with the two different sets of layers highlighted in orange and dark blue; and d) conformation of one BBDDADC linker in the framework. Color code: carbon—gray, nitrogen—blue, oxygen—red, zinc—light blue, and hydrogen—light gray. Topological representation: green squares—simplified Zn_2_(CO_2_)_4_‐cluster; two connected red triangles—simplified BBDDADC linker.

DUT‐184 ([Zn_2_(BBDDADC)(NMP)_2_]_*n*_) crystallizes as yellow needle‐shaped single crystals. The crystal structure of DUT‐184 was determined by single‐crystal X‐ray diffraction (SC XRD), indicating that the MOF crystallizes in the orthorhombic space group *Cccm* with unit cell parameters *a* = 15.788 Å, *b* = 37.045 Å, and *c* = 12.901 Å. The resulting structure has the composition [Zn_2_(BBDDADC)(NMP)_2_]_*n*_(L)_*x*_ (L–uncoordinated solvent molecules) and is shown in **Figure** **2**a–c. It consists of zinc paddle wheel units saturated by disordered NMP molecules.

The 3D structure of the MOF is assembled by two sets of parallel 2D layers that are catenated. These sets are parallel to the crystallographic planes (2,2,0) and (2,−2,0). The 1D channels with a diameter of around 9.0 Å (excluding van der Waals radii) are running along the *c*‐axis. Simplification of the structure using the ToposPro Software^[^ ^24^ ^]^ led to a 3,4‐c net of **bex** topology for each layer and, therefore, **bex‐c** topology for the overall network (point symbol: (4.6^2^)_2_(4^2^.6^2^.8^2^)). A detailed view of one layer (Figure S10, Supporting Information) shows that the biphenyl bridge of the linker is parallel to the plane of the layer, while the dibenzoazepine unit and connected carboxylates are oriented out of the plane. Looking at a single linker, it is noticeable that the aliphatic parts of the seven‐membered rings of both sides of the linker are oriented toward opposite directions regarding the biphenyl moiety and the angle between the two carboxylates of the dibenzoazepine units is exceptionally small with the value of 107.79° (Figure 2d).

The comparable tetratopic BenzTB linker in combination with paddle wheel units (in DUT‐10–DUT‐12)^[18a]^ shows angles of around 120° and the BBCDC linker (in DUT‐49)^[17a,17b]^ shows 90° angles, which unexpectedly places the angle of the seven‐membered ring 5,5 15 0‐([1,1 0‐biphenyl]‐4,4 0‐diyl)bis(10,11‐dihydro‐5H‐dibenzo[b,f]azepine‐2,8‐dicarboxylate (BBDDADC) linker in DUT‐184 between a five‐membered ring and unconnected benzyl rings.

Supercritical CO_2_ activation of DUT‐184 led to the loss of long‐range order according to powder X‐ray diffraction (PXRD) (Figure S13a, Supporting Information). Thermogravimetric analysis after activation indicates that the coordinated NMP molecules were removed, which could be the reason for the loss of crystallinity. Nitrogen physisorption at 77 K (Figure S13b, Supporting Information) shows a low uptake of 2.69 mmol g^−1^.

### 2.3 DUT‐193 Structure and Porosity

**Figure 3:**
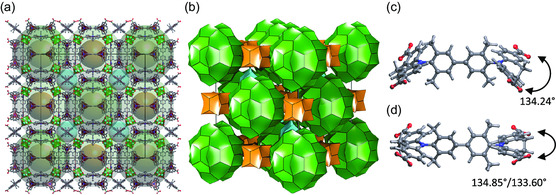
a) Structure and underlying augmented net of DUT‐193 with three types of pores; b) natural tiling of DUT‐193; c,d) the two linker conformations with their CO_2_H–N–CO_2_H angles. Color code: carbon—gray, nitrogen—blue, oxygen—red, zinc—light blue, and hydrogen—light gray/ or omitted. Topological representation: green octahedra—simplified Zn_4_O(CO_2_)_6_‐cluster; two connected red triangles—simplified TBBDDADC linker. Pores: rhombicuboctahedral mesopore—green, cubic micropore—orange, and tetrahedral micropore—light blue.

DUT‐193 ([Zn_4_O(TBBDDADC)_3/2_]_*n*_) was synthesized under similar conditions, but the sterically hindered torsion of azepine units in the linker favored the crystallization of a different phase, macroscopically appearing as yellow octahedra instead of needles. Structure determination from synchrotron SC XRD data collected on a solvated crystal could identify the cubic *F23* space group and the unit cell with *a* = 57.95 Å. Ab initio solution of the crystal structure provided the position of disordered Zn_4_O^6+^ clusters (Figure S15, Supporting Information). Because of the observed disorder of the cluster and low data quality, the position of ligand molecules could not be derived from the least‐square refinement of the structural model. Therefore, we applied a modeling approach to obtain the complete structural model of the 3D framework, which was used for Rietveld refinement (Figure S16, Supporting Information), confirming the correctness of the average crystal structure (**Figure** **3**a). The resulting structure contains two geometrically independent linkers (Figure 3c,d). Even though the framework composition is similar to that of DUT‐13 and DUT‐190,[^18^, ^25^] due to larger angles between carboxylate groups in the linkers, the resulting framework has an entirely different topology. The angles ranging from 133.60° and 134.85° are much bigger than the ones observed for the BBDDADC linker incorporated into DUT‐184 (a direct comparison of the linkers is shown in Figure S12, Supporting Information). One of the main reasons for the formation of different structures is the functionalization of the biphenyl moiety by four methyl groups in 3,3′‐ and 5,5′‐positions, which hinders the rotational motions of dihydro azepine moieties and forces the arrangement in defined conformations. Successively, the conformation of dihydro azepine defines the energetically favorable geometrical configuration of carboxylate groups, which is often one of the main factors, influencing the formation of particular framework topologies. Simplification of the MOF structure by all node approach and following rules described by O'Keeffe et al.[^20^, ^21^] using the ToposPro software^[^ ^24^ ^]^ led to a so‐far‐unknown cubic topology with the point symbol (5^2^.6)_3_(5^3^)_3_(5^6^.6^6^.8^3^)_2_ that will be further denoted with the new RCSR symbol **bff**. The topological representation of the net can be seen in Figure S19, Supporting Information.

The construction of the natural tiling with the help of the *3dt* software^[^ ^26^ ^]^ allows detailed insight into the trimodal pore system of DUT‐193 (Figure 3b). The largest pore is confined by 24 Zn_4_O^6+^ clusters, resembling the corners of a rhombicuboctahedron. Both linker conformations are part of this pore, with the one shown in Figure 3c being parallel to the unit cell faces and the one shown in Figure 3d being parallel to the (110), (101), or (011) planes, respectively. This mesopore has a geometrical diameter of 30.3 Å, taking the van der Waals radii of the atoms into account. In the unit cell, it is located at the face centers and corners, which is characteristic of the face‐centered space group. A smaller pore of 12.2 Å diameter is built by eight Zn_4_O^6+^ clusters connected by six linkers of the conformation shown in Figure 3c. It has a cubic shape and is located at the center of the unit cell and the center of all unit cell edges. Along each cell axis, rhombicuboctahedral and cubic pores alternate. The smallest pore has a geometrical diameter of 8.6 Å and is confined by four zinc clusters connected by four outer azepinedicarboxylate units of the second linker conformation (Figure 3d), leading to a tetrahedral shape. These tetrahedral pores fill the remaining space in the unit cell and occur twice on each space diagonal. A detailed view of the pores is shown in Figure S17, Supporting Information.

**Figure 4:**
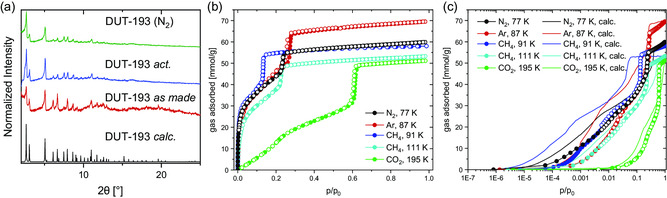
a) PXRD patterns of DUT‐193 calculated from the structure (black), as made (red), activated (blue), and after nitrogen physisorption (green); b) physisorption of several gases on DUT‐193 at different temperatures stated in the legend (filled symbols–adsorption; empty symbols–desorption); and c) semilogarithmic plot of the same physisorption experiments combined with calculated adsorption isotherms (lines).

PXRD patterns (**Figure** **4**a) showed that after desolvation using supercritical CO_2,_ the structure of the solvated phase is retained. Likewise, after nitrogen adsorption at 77 K, changes can neither be observed in PXRD nor in scanning electron microscopy (SEM) images (Figure S18, Supporting Information). The PXRD of the synthesized bulk material fits very well with the PXRD pattern calculated from modeled structure obtained by Rietveld refinement and SC XRD.

Nitrogen physisorption at 77 K (Figure 4b) shows a saturation uptake of 59.87 mmol g^−1^ resulting in a high experimental pore volume (PV) of 2.076 cm^3^ g^−1^ (*p*/*p*_*0*_ = 0.992) slightly below the calculated value of 2.1 cm^3^ g^−1^, obtained using the Zeo++ software.^[^ ^27^ ^]^ The PV of DUT‐193 is comparable to that of DUT‐13 (2.03 cm^3^ g^−1^)^[^ ^28^ ^]^ and lower than that of DUT‐49 (2.91 cm^3^ g^−1^).^[17b]^ Interestingly, the pore diameter of the largest pores of these MOFs is far lower than the diameter of the DUT‐193 mesopore that can be obtained from the pore size distribution (≈30 Å, Figure S22, Supporting Information).[^17^, ^18^] Even DUT‐25, where an additional tritopic linker is added to the BenzTB linker, only has an ellipsoidal mesopore that can slightly exceed the diameter of the nearly spherical mesopore of DUT‐193 in 1D.^[18c]^ Therefore, DUT‐193 has the biggest spherical mesopore upon MOFs of comparable linkers.

In the nitrogen physisorption at a relative pressure of 0.26, a saturation is reached. In the relative pressure range of 0.05–0.20, a linear region of the Brunauer–Emmett–Teller (BET) plot suitable for the calculation of the SA is observed. Fulfilling all three consistency criteria,^[^ ^29^ ^]^ an experimental BET area of 3288 m^2^ g^−1^ (*C* = 70) can be determined (Figure S21, Supporting Information), which is lower compared to the geometrically calculated value of 4480 m^2^ g^−1^. Since the BET model in a strict sense is applicable to micropores and small mesopores only to a limited extent, the geometrically calculated value in this case is more reliable.[^29^, ^30^] After this linear range, a steep step in the isotherm is observed, corresponding to the filling of mesopores present in the structure.

The adsorption behavior of DUT‐193 was further studied by grand canonical Monte Carlo (GCMC) simulations performed on an MOF with fixed atom positions. An excellent agreement between the experimental and calculated adsorption isotherms for the four gases could be achieved, as evidenced from Figure 4c.

Since nitrogen physisorption shows no hysteresis between adsorption and desorption branches and considering the similarity of the linker with those in DUT‐49 and DUT‐13 guest responsive frameworks, physisorption experiments using methane and carbon dioxide have been conducted (Figure 4b). Because of high adsorption enthalpies, known for methane and carbon dioxide, guest‐responsive properties of DUT‐193 can be evaluated. In addition to adsorption measurements of these gases at their corresponding boiling points, methane adsorption at 91 K was measured to further increase host–guest interactions.^[^ ^31^ ^]^ Interestingly, only for the adsorption of argon, a small hysteresis could be observed, leading to the conclusion that no major structural changes seem to occur during the adsorption/desorption processes. Neither the strongly interacting CO_2_ molecules nor methane adsorption at 91 K does induce a transformation classifying DUT‐193 as a rigid MOF of the second generation.^[^ ^32^ ^]^ Up to 69.48 mmol g^−1^ argon could be adsorbed on DUT‐193 at 87 K. At 111 K, 53.26 mmol g^−1^ methane and at 91 K, 58.02 mmol g^−1^ methane are adsorbed. Finally, at 195 K, the adsorption of 51.13 mmol g^−1^ CO_2_ is observed. The noticeable shift of the mesopore‐filling step to higher relative pressures for the CO_2_ adsorption can be explained by changed guest–guest interactions below the triple point (216.5 K, 0.51 MPa) of CO_2_. A similar phenomenon has been observed in other mesoporous frameworks and can be explained by uncertainty in calculation of *p*/*p*_0_ below the triple point of CO_2_.[^17^, ^33^]

**Figure 5:**
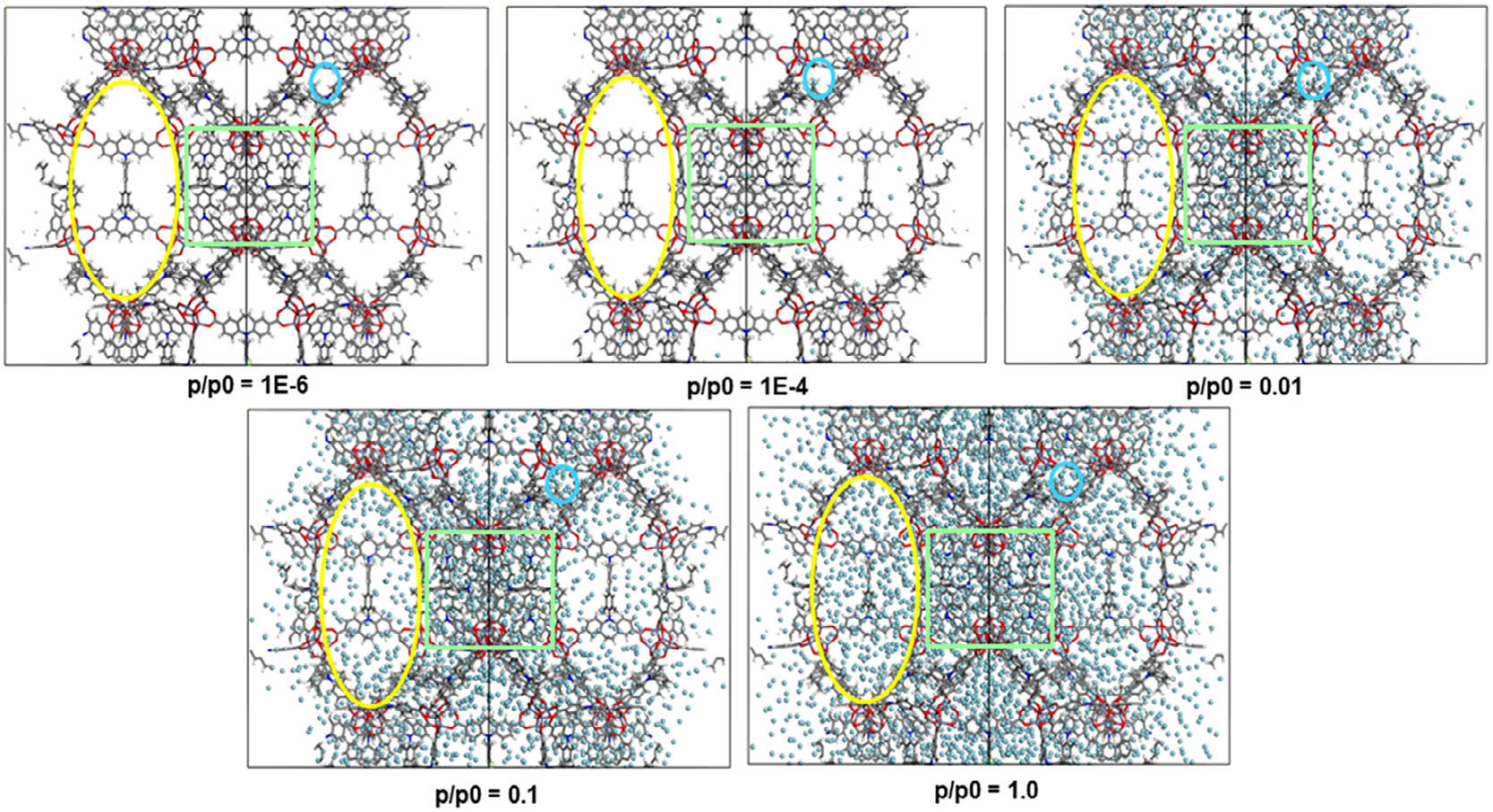
Illustrative grand canonical Monte Carlo configurations of Ar adsorption in DUT‐193 at 87 K with *p*/*p*_0_ ranging from 10^−6^ to 1 bar: tetrahedral pores, cubic central pores, and large pores are indicated in blue, green, and yellow, respectively.

Analysis of the GCMC simulations conducted for all four gases at different pressures further gave insight into the microscopic adsorption mechanism in DUT‐193. The adsorption of Ar proceeds sequentially in the different pores of DUT‐193 (**Figure** **5**): 1) at very low pressure, small tetrahedral pores (blue color) start to be populated by the guest molecules; 2) at moderate pressure, the cubic central pores are filled, and the large pores start to be occupied; and finally 3) at high pressure, we observe a filling of all the tetrahedral, cubic, and large pores. Similar adsorption behavior was found for the other three guest molecules N_2_, CH_4_, and CO_2_ as depicted in Figure S24–S26, Supporting Information.

Thus, by the introduction of a seven‐membered ring and methyl groups into the biphenyl bridge of the linker, the principle of geometry mismatch successfully led to a new topological arrangement of tetratopic linkers and Zn_4_O^6+^ clusters. The new and highly symmetric **bff** topology does not allow framework flexibility, because the torsion of the biphenyl moiety is sterically hindered, resulting in two geometrically independent linker conformations in the structure. Therefore, unlike all the other linkers shown in Figure 1, the TBBDDADC linker leads to a rigid framework.

## 3 Conclusion

The geometry mismatch synthetic strategy, utilizing new tetratopic linkers containing seven‐membered ring (azepine) units, resulted in two new MOF compounds. Utilization of the new H_4_BBDDADC linker thereby led to a 3D framework that is built of catenated Zn_2_(BBDDADC) 2D layers. Surprisingly, the interconnecting of peripheral phenyl rings of the linker by −CH_2_−CH_2_− bridge resulted in very low angles of 108° between carboxylates, even though a seven‐membered ring was expected to push the angles to higher values.

The introduction of additional methyl groups in the biphenyl bridge of the H_4_TBBDDADC linker not only introduced a twist between the azepine units and the bridge of the linker, but also led to a different framework with much higher carboxylate to carboxylate angles of 134° on average. This indicates certain structural flexibility of linkers including such seven‐membered rings. The resulting rigid DUT‐193 framework is based on Zn_4_O^6+^ octahedral nodes, interconnected by TBBDDADC^4−^ linkers, rewards us with the successful discovery of the new **bff** topology that is based on trigonal and octahedral nodes. The trimodal pore system contains a large mesopore with a diameter of 30.3 Å (taking van der Waals radii into account), the largest mesopore size observed in all MOFs built of the tetratopic linkers with comparable linear dimensions, geometry, and coordination ability (Figure 1).[^17^, ^18^] The adsorption data obtained for the new highly symmetrical cubic DUT‐193 showed good agreement between experimentally measured and theoretically calculated isotherms and high porosity for N_2_ (59.87 mmol g^−1^), Ar (69.48 mmol g^−1^), CH_4_ (53.26 mmol g^−1^), and CO_2_ (51.13 mmol g^−1^) at their corresponding boiling points. Additionally, GCMC calculations reveal the microscopic adsorption behaviors of the gases, first adsorbing in the smallest tetrahedral micropores, then in the cubic micropores and walls of the large rhombidodecahedral mesopore followed by a final filling of all pores at higher pressures.

Combining the two new linkers with other metal clusters, especially of higher connectivity, such as zirconium‐based clusters, may lead to chemically stable highly porous MOFs possessing new framework topologies. Generally, the introduction of seven‐membered rings into linkers could lead to a certain control of conformational freedom opening a wider and interesting field for the design of new MOFs.

## 4 Experimental Section

4.1

4.1.1

##### General Methods

All chemicals and solvents used in the syntheses were at least of reagent grade and were used without further purification.

^1^H and ^13^C nuclear magnetic resonance (NMR) spectra were acquired on a Bruker Avance III 500 spectrometer (500 and 125.8 MHz for ^1^H and ^13^C, respectively) or a Bruker Avance III HD 300 (300 and 75.5 MHz for ^1^H and ^13^C, respectively). All ^1^H and ^13^C NMR spectra are reported in parts per million (ppm) downfield of tetramethylsilane and were measured relative to the residual signals of the solvents at 7.26 ppm (CDCl_3_), 5.32 ppm (dichloromethane‐d_2_ (dichloromethane = DCM)), or 2.50 ppm (dimethyl sulfoxide‐d_6_ (dimethyl sulfoxide = DMSO))^[^ ^34^ ^]^ Additionally, all spectra, including assignments of all signals, can be found in the Supporting Information.

Attenuated total reflection infrared (ATR‐IR) spectra were recorded in a wavenumber range of 4000–600 cm^−1^ using a BRUKER Vertex 70 IR spectrometer. All samples were placed on the ATR crystal in the solid state. For all bands, the corresponding wave numbers were given as well as indication of the intensity in brackets as w—weak, m—medium, or s—strong. All spectra can be found in Figure S9, Supporting Information.

Elemental Analysis (C, H, N) was performed using a Vario MICRO Cube Elementar device.

PXRD patterns were collected in transmission geometry with a STOE STADI P diffractometer, equipped with MYTHEN 100 K (DECTRIS) 1D detector, and operated at 40 kV and 30 mA with monochromatic Cu‐Kα_1_ (*λ* = 0.15405 nm) radiation, a scan speed of 120 s step^−1^ and a detector step size of 2*θ* = 6°. All samples were prepared using the procedure developed for air/moisture‐sensitive samples and measured in a flatbed sample holder. “As made” samples were analyzed while suspended in *N*,*N*‐dimethylformamide (DMF). Desolvated samples were handled in a glove box under argon atmosphere. Theoretical PXRD patterns were calculated based on crystal structures using Mercury 4.0 software package.^[^ ^35^ ^]^

Thermogravimetric (TGA) and differential thermal analyses (DTA) were carried out in air using a Netzsch STA 409 C/CD thermal analyzer with a heating rate of 5 K min^−1^.

SEM images were taken with secondary electrons in a HITACHI SU8020 microscope using 2.0 kV acceleration voltage at different working distances and different magnifications. The samples were prepared on a sticky carbon sample holder. To avoid degradation upon exposure to air, the samples were prepared in a glove box under argon atmosphere. All samples were sputtered with gold in argon atmosphere before measurement.

Physisorption of N_2_ (99.999%), Ar (99.999%), CH_4_ (99.999%), and CO_2_ (99.9995%) was performed on a *BELSORP‐max* instrument of *MICROTRAC MRB*. As equilibrium conditions, a pressure change of 1% upon 300 s was chosen for each point of the isotherm. The dead volume was determined using helium (99.999%). The values for adsorbed gases are given at standard conditions (273.15 K, 101.325 kPa) in mmol g^−1^. To reach the corresponding adsorption temperatures, a DE‐202AG (ARS‐Cryo) cryostat with connected helium cycle and an LS‐366 (LAKE SHORE) control unit was used. A water‐cooled ARS‐2HW helium compressor dissipated produced heat of the cryostat. The sample was placed in a self‐made 3 cm long, cylindrical copper cell with 1 cm diameter closed by a copper dome, isolated by dynamic vacuum (*p* < 10^−4^ kPa), and connected to the instrument using a 3 mm stainless steel capillary.

Nitrogen physisorption on DUT‐184 was measured at 77 K on a QUANTACHROME Quadrasorb SI automated SA and pore size analyzer.

##### Single‐Crystal XRD for DUT‐193 and DUT‐184

A single crystal of DUT‐193 was prepared in a borosilicate glass capillary (*d* = 0.3 mm) with a small amount of solvent. The data set was collected at the BL14.2 beamline of the BESSY II synchrotron, operated by Helmholtz–Zentrum Berlin für Materialien and Energie.^[^ ^36^ ^]^ Four images from different crystal orientations were collected to determine the crystal symmetry and scan angle range using the iMosflm program.^[^ ^37^ ^]^ The φ scan with an oscillation step of *Δφ* = 0.1° was used for the collection of 1800 frames, which were processed automatically using XDSAPP 3.0 software.^[^ ^38^ ^]^ The crystal structure was solved by direct methods in the *F*432 space group resulting in an incomplete model containing disordered Zn_4_O clusters. Least‐squares refinement of the structural model on *F* ^2^ using the SHELX‐2018/3 program package^[^ ^39^ ^]^ did not improve the model, e.g., no reasonable positions for light atoms (C, N, O) could be identified. However, the position of the cluster was sufficient to construct a chemically reasonable ordered structural model of DUT‐193 in the *F*23 space group using Materials Studio 5.0 software.^[^ ^40^ ^]^ Reducing the space group symmetry was required to avoid structural disorder in the average structural model, which was further used in Rietveld refinement. The average structural model of DUT‐193, created using the visualization module of Materials Studio 5.0, was further subjected to Rietveld refinement using the Reflex tool.^[^ ^40^ ^]^ The PXRD profile was refined using a Pawley fit, followed by a Rietveld refinement of the crystal structure. Zinc atoms, carboxylate and phenyl groups of the ligand molecules were handled as rigid bodies in the refinement to reduce the number of parameters. The Rietveld plot is given in Figure S16, Supporting Information. CCDC‐2268284 contains the supplementary crystallographic data for DUT‐193. This data can be obtained free of charge from the Cambridge Crystallographic Data Centre via www.ccdc.cam.ac./structures.uk

##### Structural Data for DUT‐193

C_144_H_108_N_6_O_26_Zn_8_, M = 2861.49 g mol^−1^, cubic, *F23* (no. 196), *a* = 57.5042(5) Å, *V* = 190151.0(5) Å^3^, *Z* = 16, *λ* = 1.54059 Å, *T* = 296 K, 2*θ*_range_ = 3°–50°, profile function Thompson‐Cox‐Hastings, *U* = 0.06248, *V* = −0.03855, *W* = 0.00862, *X* = 0.07358, *Y* = 0.14232, refined motion groups/degree of freedom 53/53, *R*_p_ = 0.0849, and *R*_wp_ = 0.1236.

A single crystal of as‐synthesized DUT‐184 was prepared in a nylon cryoloop with paratone oil. The diffraction images were collected at 250 K using a Bruker KAPPA APEX2 diffractometer, equipped with a long focus X‐ray tube with Mo‐anode and graphite monochromator (*λ* = 0.71073 Å) and an APEX2 CCD detector. The unit cell parameters were determined based on three short scans performed on different crystal orientations. The data collection strategy was optimized using the APEX3 software, and collected images were integrated using the SAINT software. Further instrument‐ and sample‐related intensity corrections were applied using the SADABS software. The crystal structures were solved by direct methods implemented in the SHELXS‐2018/3 program.^[^ ^39^ ^]^ The obtained model was refined by full matrix least squares on *F* ^2^ using SHELXL‐2018/3.^[^ ^39^ ^]^ All non‐hydrogen atoms were refined in the anisotropic approximation. Hydrogen atoms were refined in geometrically calculated positions using a ‘‘riding model’’ with *U*_iso_(H) = 1.2*U*_iso_(C). The disorder of C8 atoms of hydroazepine ring and C10 and C11 atoms of phenyl ring was resolved by splitting on two symmetrically dependent positions, each refined with occupancies of 50%. The positions of disordered solvent molecules in the pores could not be determined from the Fourier electron density maps, and therefore, the SQUEEZE routine was applied to reduce the contribution of the solvent molecules to the structure factors.^[^ ^41^ ^]^ As a result, 1408 electrons were removed from 3219 Å^3^ of solvent‐accessible voids of DUT‐184 structure. CCDC‐2268285 contains the supplementary crystallographic data for DUT‐184. This data can be obtained free of charge from the Cambridge Crystallographic Data Centre via https://www.ccdc.cam.ac.UK/data_request/cif.

##### Structural Data for DUT‐184

C_54_H_46_N_4_O_10_Zn_2_ × *n*NMP, *M* = 1041.69 g mol^−1^, orthorhombic, *Cccm* (no. 66), *a *= 15.7880(16) Å, *b *= 37.045(3) Å, *c* = 12.9010(10) Å, *V *= 7545.3(11) Å^3^, *Z *= 4, *λ* = 0.71073 Å, *T *= 250 K, *θ*_max_ = 25.2°, reflections/parameter/restraints 3523/208/49, *R*_int_ = 0.1582, *R*_1_ = 0.0487 (*I* > 2*σ*), *wR*_2_ = 0.1662 (all data), *S* = 1.023 (all data) largest diff. peak 0.419 e Å^−3^, and hole −0.380 e Å^−3^.

##### Synthesis Procedures

*Synthesis of 2,8‐Dibromo‐10,11‐dihydro‐5H‐dibenzo[b,f]azepine*: for this reaction, a procedure reported by Smith et al. was used.^[^ ^42^ ^]^ Therefore, 82.0 g (1.36 mol, 68.01 eq.) silica gel was dried at 120 and 100 mbar for 1 h. After cooling down, 4.04 g (97%, 20.07 mmol, 1.00 eq.) 10,11‐dihydro‐5*H*‐dibenzo[*b*,*f*]azepine and 400 mL dry DCM were added to obtain a white suspension. Separately, 7.24 g (99%, 40.27 mmol, 2.01 eq.) *N*–bromosuccinimide was dissolved in 250 mL dry DCM. The solution was added to the suspension at room temperature over a period of 90 min. After stirring overnight, the dark‐green suspension was filtered. The filtrate was concentrated to 100 mL and washed with deionized water three times. The organic phase was dried using MgSO_4_, filtered and then the solvent was evaporated. The crude product was recrystallized using 30 mL chloroform. The 4.10 g (58% yield) off‐white needles of the product were obtained. ^1^H NMR (300 MHz, DCM‐d_2_) *δ* (ppm) = 7.18 (s, 2H), 7.16 (m, 2H), 6.65 (d, *J *= 9.1 Hz, 2H), 6.10 (s, 1H), and 3.01 (s, 4H).

*Synthesis of 10,11‐Dihydro‐5H‐dibenzo[b,f]azepine‐2,8‐dicarbonitrile*: As the work with cyanides is extremely dangerous, a solution for the deactivation consisting of 5% hydrogen peroxide and concentrated ammonia at a pH of at least 11 was prepared beforehand. The solution was used to deactivate all remaining cyanide solutions and the devices that were exposed to cyanides. In a pear‐shaped flask, 4.97 g (14.08 mmol, 1.00 eq.) 2,8‐dibromo‐10,11‐dihydro‐5*H*‐dibenzo[*b*,*f*]azepine and 38.6 mg (97%, 67.5 μmol, 0.48 mol%) 1,1‐bis(diphenylphosphino) ferrocene (dppf) were dissolved in 15 mL dry DMF. In parallel, 36.6 mg (560 μmol, 0.04 eq.) zinc powder, 31.8 mg (97%, 33.8 μmol, 0.24 mol%) Pd_2_(dba)_3_, and 124 mg (565 μmol, 0.04 eq.) zinc acetate dihydrate were prepared in a Schlenk flask. After the addition of 2.54 g (98%, 21.22 mmol, 1.5 eq.) to the Schlenk flask, it was evacuated and flushed with argon several times and 5 mL DMF was added. Then the solution of the starting material and dppf was added to the cyanide suspension. The reaction mixture was stirred at 80 °C for 8 d and was then quenched using 80 mL of NH_3_/NH_4_Cl buffer. The precipitate was filtered off and washed with 40 mL of the buffer solution three times first and then washed with water, followed by ethanol and, after that, toluene. After drying in an oven at 80 °C, 3.12 g (98% purity, 89% yield) of a bright yellow powder was obtained. ^1^H NMR (300 MHz, DMSO‐d_6_) *δ* (ppm) = 9.52 (s, 1H), 7.54 (s, 2H), 7.53 (dd, *J *= 8.3 Hz, *J *= 2.0 Hz, 2H), 7.15 (d, *J *= 8.3 Hz, 2H), and 2.99 (s, 4H).

*Synthesis of 10,11‐Dihydro‐5H‐dibenzo[b,f]azepine‐2,8‐dicarboxylic acid (H*_*2*_ *DDADC)*: A solution of 7.04 g (98%, 17.25 mmol, 17.92 eq.) sodium hydroxide and 22.5 mg (118.1 μmol, 1.23 mol%) copper(I)‐iodide in 76 mL deionized water was prepared and added to a 250 mL round bottom flask with 2.41 g (98%, 9.62 mmol, 1.00 eq.) 10,11‐dihydro‐5*H*‐dibenzo[*b*,*f*]azepine‐2,8‐dicarbonitrile. The suspension was stirred for 7 d and filtered using Celite. The goldish‐brown filtrate was acidified using 6 m HCl until pH 2 was reached. The yellowish precipitate was filtered off and washed with water until pH 6 was reached. After drying at 80 °C, the raw product was suspended in 200 mL methanol, filtered again, and washed thoroughly with additional methanol to obtain a pure product from the filtrate after complete evaporation. And, 1.44 g (41% yield) of a yellow powder were obtained. ^1^H NMR (300 MHz, DMSO‐d_6_) *δ* (ppm) = 12.39 (s, 2H), 9.27 (s, 1H), 7.66 (m, 4H), 7.09 (d, *J* = 9.1 Hz, 2H), and 3.02 (s, 4H). IR υ cm^−1^ = 3401 (m), 3357 (w), 3035 (w), 2958 (m), 2931 (m), 2871 (w), 2856 (w), 2663 (w), 2561 (m), 1676 (s), 1609 (m), 1594 (s), 1501 (s), 1424 (m), 1405 (m), 1351 (m), 1323 (m). 1284 (s), 1233 (s), 1167 (m), 1138 (m), 1115 (m), 950 (w), 915 (w), 824 (w), 765 (m), 750 (w), 728 (w), 657 (w), 650 (w), 607 (w).

*Synthesis of dibutyl 10,11‐Dihydro‐5H‐Dibenzo[b,f]azepine‐2,8‐dicarboxylate (Bu*_*2*_ *DDADC)*: In a 100 mL round bottom flask, 1.40 g (4.94 mmol, 1.00 eq.) H_2_DDADC was suspended in 75 mL (60.75 g, 819.58 mmol, 165.91 eq.) 1‐butanol and 300 μL (549 μg, 5.6 μmol, 0.11 mol%) sulfuric acid was added. After stirring at 120 °C for 4 d, the remaining butanol was evaporated. The raw product was dissolved in chloroform and extracted three times with a saturated potassium carbonate solution and three times with water. The organic phase was dried using MgSO_4_ and filtered, and then the remaining chloroform was evaporated. The reddish‐brown crude product was then recrystallized from a 1:3 mixture of ethyl acetate and *n*‐pentane or purified by column chromatography using a solvent mixture of 1:4 (ethyl acetate/*iso*‐hexane). *R*_f_(TLC) = 0.75 in EtOAc/*i*Hex 1:4. ^1^H NMR (300 MHz, CDCl_3_) *δ* (ppm) = 7.78 (dd, *J *= 1.8 Hz, *J *= 9.0 Hz, 2H), 7.77 (s, 2H), 6.78 (d, *J *= 9.0 Hz, 2H), 6.63 (s, 1H), 4.29 (t, *J *= 6.6 Hz, 4H), 1.74 (dt, *J *= 14.7 Hz, *J *= 6.7 Hz, 4H), 1.47 (dq, *J *= 14.5, *J* = 7.3 Hz, 4H), 0.98 (t, *J *= 7.4 Hz, 6H). ^13^C NMR (76 MHz, CDCl_3_) *δ* (ppm) = 166.62, 145.23, 132.79, 128.92, 128.23, 122.03, 118.17, 64.65, 35.24, 31.03, 19.45, 13.94. Elemental analysis of C_24_H_29_N_1_O_4_, calculated: C 72.89%, H 7.39%, N 3.54%; found: C 72.47%, H 7.26%, N 3.47%. IR υ cm^−1^ = 3343 (m), 3230 (w), 3208 (w), 3135 (w), 3126 (w), 3119 (w), 2958 (m), 2933 (m), 2890 (m), 2874 (m), 2867 (m), 2628 (w), 2566 (w), 1704 (s), 1670 (s), 1620 (m), 1597 (m), 1528 (m), 1502 (m), 1472 (m), 1459, 0255 (w), 1443 (w), 1421 (m), 1392 (w), 1361 (m), 1333 (m), 1290 (s), 1282 (s), 1265 (m), 1232 (s), 1207 (m), 1195 (m), 1169 (m), 1144 (m), 1114 (m), 1105 (m), 1064 (w), 1033 (m), 997 (w), 983 (m), 949 (w), 928 (w), 918 (w), 888 (w), 841 (w), 824 (w), 805 (w), 767 (m), 740 (w), 709 (w), 684 (w), 664 (w), 648 (w), 607 (w).

*Synthesis of 4,4′‐Dibromo‐3,3′,5,5′‐tetramethyl‐1,1′‐biphenyl*: For this reaction, a procedure reported by Sasaki et al. was used.^[^ ^43^ ^]^ The 4.41 g (31.91 mmol, 1.02 eq.) potassium carbonate, 5.10 g (99%, 15.65 mmol, 0.50 Äq.) tetrabutylammonium bromide, and 355.5 mg (99%, 1.57 mmol, 0.05 eq.) Pd(OAc)_2_ were submitted to a 25 mL Schlenk flask. Using syringes, 10 g (97%, 31.19 mmol, 1.00 eq.) 2‐bromo‐5‐iodo‐1,3‐dimethylbenzene, 3.6 mL (3.40 g, 46.49 mmol, 1.49 eq.) DMF, 1.4 mL (1.40 g, 77.98 mmol, 2.50 eq.) deionized water, and 0.38 mL (299 μg, 4.97 mmol, 0.16 eq.) *iso*‐propanol were added successively. The mixture was stirred overnight at 115 °C. After cooling down, diethyl ether was added, and the black suspension was filtered. The filtrate was extracted three times using brine. The brown solution was dried using MgSO_4_, filtered and the remaining solvent was evaporated. The crude product was purified using column chromatography with pure *iso*‐hexane. And, 3.40 g (59% yield) of a white powder was obtained. *R*_f_(TLC) = 0.79 in 100% *i*Hex. ^1^H NMR (300 MHz, DCM‐d_2_) *δ* (ppm) = 7.29 (s, 4H), 2.47 (s, 12H).

*Synthesis of Tetrabutyl 5,5′‐(3,3′,5,5′‐tetramethyl‐[1,1′‐biphenyl]‐4,4′‐diyl)bis(10,11‐dihydro‐5H‐dibenzo[b,f]azepine‐2,8‐dicarboxylate) (Bu*_*4*_ *TBBDDADC)*: In a Schlenk flask, 527.7 mg (97%, 3.06 mmol, 3.03 eq.) LiHMDS, 93.5 mg (95%, 104 μmol, 0.10 eq.) RuPhos‐Pd‐G4, 48.2 mg (98%, 101 μmol, 0.10 eq.) RuPhos, and 372.1 mg (1.01 mmol, 1.00 eq.) 4,4′‐dibromo‐3,3′,5,5′‐tetramethyl‐biphenyl were dissolved in 10 mL dry and degassed 1,4‐dioxane under argon. In parallel, 998.9 mg (2.52 mmol, 2.50 eq.) Bu_2_DDADC was dissolved in 12 mL dry and degassed 1,4‐dioxane under argon. After heating the solution with the catalyst to 100 °C, the solution of Bu_2_DDADC was added, and the resulting suspension was refluxed for 11 d. The cooled‐down reaction mixture was filtered using silica and thoroughly washed with DCM. The solvents of the obtained filtrate were evaporated, and the crude product was purified using column chromatography with a solvent mixture of chloroform/DCM/ethyl acetate (1:1:0.04). And, 331.5 mg (33% yield) of the glassy yellow product could be obtained. *R*_f_(TLC) = 0.51 in CHCl_3_/DCM/EtOAc 1:1:0.04. ^1^H NMR (300 MHz, DCM‐d_2_) *δ* (ppm) = 7.80 (d, *J* = 2.2 Hz, 4H), 7.60 (s, 4H), 7.57 (dd, *J* = 8.9 Hz, *J* = 2.2 Hz, 4H), 6.44 (d, *J* = 8.9 Hz, 4H), 4.25 (t, *J* = 6.6 Hz, 8H), 3.27 (s, 8H), 2.08 (s, 12H), 1.72 (dt, *J* = 14.5 Hz, *J* = 6.7 Hz, 8H), 1.46 (td, *J* = 14.8 Hz, *J* = 7.4 Hz, 8H), 0.96 (t, *J* = 7.4 Hz, 12H). ^13^C NMR (76 MHz, DCM‐d_2_) *δ* (ppm) = 166.56, 147.46, 143.43, 140.49, 137.99, 132.48, 132.25, 128.96, 128.57, 122.38, 119.72, 64.78, 38.36, 31.27, 19.70, 18.08, 13.95. Elemental analysis of C_64_H_72_N_2_O_8_, calculated: C 77.08%, H 7.28%, N 2.81%; found: C 77.62%, H 7.88%, N 2.43%. IR υ cm^−1^ = 2957 (m), 2930 (m), 2871 (m), 1705 (s), 1612 (w), 1600 (m), 1565 (w), 1490 (m), 1464 (w), 1451 (w), 1416 (w), 1385 (w), 1321 (w), 1303 (w), 1276 (s), 1252 (s), 1231 (w), 1211 (m), 1191 (m), 1142 (m), 1130 (m), 1117 (w), 1085 (w), 1060 (w), 1037 (w), 1017 (w), 1007 (w), 992 (w), 969 (w), 956 (w), 942 (w), 917 (w), 903 (w), 879 (w), 867 (w), 824 (w), 768 (m), 740 (w), 732 (w).

*Synthesis of 5,5′‐(3,3′,5,5′‐Tetramethyl‐[1,1′‐biphenyl]‐4,4′‐diyl)bis(10,11‐dihydro‐5H‐dibenzo[b,f]azepine‐2,8‐dicarboxylic acid) (H*_*4*_ *TBBDDADC)*: The 240.4 mg (95%, 243 μmol, 1.00 eq.) Bu_4_TBBDDADC was dissolved in 16 mL THF. 268.6 mg (85.7%, 4.10 mmol, 16.91 eq.) potassium hydroxide and 1.85 mL deionized water were added. The reaction mixture was refluxed for 7 d at approximately 80 °C. Additional water was added during that time to dissolve, eventually precipitating intermediate products. For the workup, remaining THF was evaporated, and solid impurities were filtered off. The filtrate was acidified using 1 m HCl until pH 2 was reached, and the precipitation of the product was complete. The yellow precipitate was filtered off and washed with water thoroughly until pH 6 of the washing solution can be reached. After drying at 80 °C 131.6 mg (76% yield), dark yellow powder could be obtained. ^1^H NMR (300 MHz, DMSO‐d_6_) *δ* (ppm) = 12.55 (s, 4H), 7.79 (s, 4H), 7.77 (d, *J* = 2.1 Hz, 4H), 7.53 (dd, *J* = 8.9 Hz, *J* = 2.1 Hz, 4H), 6.36 (d, *J* = 8.9 Hz, 4H), 3.23 (s, 8H), 2.03 (s, 12H). ^13^C NMR (76 MHz, DMSO‐d_6_) *δ* (ppm) = 166.92, 146.46, (142.65), (139.10), 137.02, 131.91, 131.80, 128.42, 128.26, (122.29), 118.79, (37.24), 17.40. Some ^13^C NMR signals could only be observed in heteronuclear single quantum correlation (HSQC) and heteronuclar multiple bond correlation (HMBC) and are shown in brackets. Elemental analysis of C_48_H_40_N_2_O_8_ · 2.8H_2_O, calculated: C 70.03%, H 5.59%, N 3.40%; found: C 70.15%, H 5.66%, N 2.97%. IR υ cm^−1^ = 3041 (m, br.), 2952 (m), 2920 (m), 2855 (m), 2644 (m), 1680 (s), 1598 (s), 1564 (m), 1489 (m), 1470 (m), 1438 (m), 1399 (m), 1379 (m), 1275 (s), 1234 (s), 1204 (s), 1128 (s), 1117 (s), 1035 (m), 986 (w), 953 (m), 918 (m), 859 (m), 826 (w), 768 (m), 724 (w), 674 (w), 648 (w), 624 (w).

*Synthesis of Tetrabutyl 5,5′‐([1,1′‐biphenyl]‐4,4′‐diyl)bis(10,11‐dihydro‐5H‐dibenzo[b,f]azepine‐2,8‐dicarboxylate) (Bu*_*4*_ *BBDDADC)*: To a Schlenk flask 474.1 mg (98%, 2.78 mmol, 2.40 eq.) LiHMDS, 20.7 mg (95%, 23.1 μmol, 2.00 mol%) RuPhos‐Pd‐G4 and 368.9 mg (98%, 1.16 mmol, 1.00 eq.) 4,4′‐dibromo‐1,1′‐biphenyl were dissolved in 10 mL dry and degassed 1,4‐dioxane under argon. In parallel, 1.0017 g (2.53 mmol, 2.19 eq.) Bu_2_DDADC was dissolved in 12 mL dry and degassed 1,4‐dioxane under argon. After heating the solution with the catalyst to 100 °C, the solution of Bu_2_DDADC was added, and the resulting suspension was refluxed for 6 d. The cooled‐down reaction mixture was filtered using silica and thoroughly washed with DCM. The solvents of the obtained filtrate were evaporated, and the crude product was purified using column chromatography with a solvent mixture of 1:1:0.08 chloroform/DCM/ethyl acetate. And, 560.7 mg (52% yield) of a bright yellow powder could be obtained. *R*_f_(TLC) = 0.74 in CHCl_3_/DCM/EtOAc 1:1:0.08. ^1^H NMR (300 MHz, DCM‐d_2_) *δ* (ppm) = 7.96 (d, *J *= 1.9 Hz, 4H), 7.90 (dd, *J *= 8.2 Hz, *J *= 2.0 Hz, 4H), 7.47 (d, *J *= 8.2 Hz, 4H), 7.31 (d, *J *= 8.8 Hz, 4H), 6.72 (d, *J *= 8.8 Hz, 4H), 4.30 (t, *J *= 6.6 Hz, 8H), 3.09 (s, 8H), 1.74 (m, 8H), 1.47 (dq, *J *= 14.5 Hz, *J *= 7.3 Hz, 8H), 0.98 (t, *J *= 7.4 Hz, 12H). ^13^C NMR (76 MHz, DCM‐d_2_) *δ* (ppm) = 166.36, 147.51, 146.91, 138.30, 132.70, 132.10, 129.83, 129.29, 128.63, 127.25, 115.17, 65.25, 31.48, 31.19, 19.68, 13.94. Elemental analysis of C_60_H_64_N_2_O_8_, calculated: C 76.57%, H 6.85%, N 2.98%; found: C 76.82%, H 6.82%, N 2.75%. IR υ cm^−1^ = 3058 (w), 3032 (w), 2958 (m), 2933 (m), 2872 (w), 1715 (s); 1676 (w), 1614 (w), 1601 (m), 1574 (w), 1495 (s), 1464 (w), 1446 (w), 1384 (w), 1354 (w), 1320 (m), 1308 (m), 1282 (m), 1259 (s), 1202 (m), 1189 (m), 1169 (m), 1113 (m), 1070 (w), 1057 (w), 1033 (w), 1018 (w), 998 (w), 967 (w), 944 (w), 913 (w), 893 (w), 842 (w), 809 (m), 787 (w), 773 (w), 763 (w), 749 (w), 713 (w), 698 (w), 671 (w), 651 (w), 642 (w).

*Synthesis of 5,5′‐([1,1′‐Biphenyl]‐4,4′‐diyl)bis(10,11‐dihydro‐5H‐dibenzo[b,f]azepine‐2,8‐dicarboxylic acid) (H*_*4*_ *BBDDADC)*: The 500.0 mg (99%, 526 μmol, 1.00 eq.) Bu_4_BBDDADC was dissolved in 50 mL THF. The 614.4 mg (85.7%, 9.38 mmol, 17.84 eq.) potassium hydroxide and 4.4 mL deionized water were added. The reaction mixture was refluxed for 5 d at approximately 80 °C. Additional water was added during that time to dissolve, eventually precipitating intermediate products. For the workup, remaining THF was evaporated, and solid impurities were filtered off. The filtrate was acidified using 1 m HCl until pH 2 was reached and the precipitation of the product was complete. The yellow precipitate was filtered off and washed with water thoroughly until pH 6 of the washing solution can be reached. After drying at 80 °C 336.4 mg (85% yield), yellow powder could be obtained. ^1^H NMR (300 MHz, DMSO‐d_6_) *δ* (ppm) = 12.95 (s, 4H), 7.92 (s, 4H), 7.84 (d, *J* = 7.6 Hz, 4H), 7.50 (d, *J* = 7.6 Hz, 4H), 7.36 (d, *J* = 7.2 Hz, 4H), 6.63 (d, *J* = 6.9 Hz, 4H), 3.03 (s, 8H). ^13^C NMR (76 MHz, DMSO‐d_6_) *δ* (ppm) = 166.78, 146.50, 146.10, 137.64, 132.28, 130.91, 129.45, 129.15, 128.27, 126.78, 114.33, 30.25. Elemental analysis of C_44_H_32_N_2_O_8_ · 1.3H_2_O, calculated: C 71.40%, H 4.71%, N 3.79%; found: C 71.32%, H 4.85%, N 3.60%. IR υ cm^−1^ = 3036 (m), 2982 (m), 2952 (m), 2930 (m), 2870 (m), 2657 (m), 2542 (m), 1686 (s), 1600 (s), 1493 (s), 1430 (m), 1413 (m), 1403 (m), 1317 (m), 1303 (m), 1284 (s), 1257 (s), 1202 (m), 1190 (m), 1171 (m), 1118 (m), 1017 (w), 993 (w), 948 (w), 914 (w), 866 (w), 838 (w), 810 (m), 768 (m), 754 (m), 730, (w), 693 (w), 651 (w), 639 (w), 615 (w), 608 (w).

*Synthesis of [Zn*_*2*_ *(BBDDADC)(NMP)*_*2*_ *]*_*n*_ *(DUT‐184)*: The 79.7 mg (305 μmol, 10.88 eq.) zinc nitrate tetrahydrate was dissolved in 4 mL NMP and 20.1 mg (28.0 μmol, 1.00 eq.) H_4_BBDDADC was dissolved in 2.7 mL NMP. After the addition of the linker solution to the zinc salt solution, the Pyrex tube was shaken and then subjected to 80 °C in an oven for 7 d. The suspension was washed thrice with fresh DMF and then solvent exchange to acetone was performed. After drying in a Jumbo Critical Point Dryer 13200J AB using supercritical CO_2,_ a yield of 20.3 mg (82.5%) could be quantified. The structure was determined using a single crystal from the mother liquor. TGA (rest mass): 19.86% ZnO. IR υ cm^−1^ = 3058 (w), 3030 (w), 2929 (w), 2857 (w), 1703 (w), 1598 (s), 1539 (m), 1493 (s), 1423 (s), 1391 (s), 1319 (m), 1286 (m), 1264 (m), 1189 (m), 1114 (m), 1017 (w), 999 (w), 951 (w), 920 (w), 890 (w), 841 (w), 815 (w), 781 (m), 755 (w), 698 (w), 674 (w).

*Synthesis of [Zn*_*4*_ *O(TBBDDADC)*_*3/2*_ *]*_*n*_ *(DUT‐193)*: The 440.3 mg (1.68 mmol, 10.08 eq.) zinc nitrate tetrahydrate was dissolved in 24 mL DMF while 119.8 mg (167 μmol, 1.00 eq.) H_4_TBBDDADC was dissolved in 16 mL NMP. The zinc salt solution was distributed equally upon four Pyrex tubes and 4 mL of linker solution was added subsequently. After short shaking, the product crystallizes for 6 d at 80 °C in an oven. The yellow octahedral crystals were washed thrice with DMF, followed by solvent exchange to acetone. After supercritical CO_2_ drying in a Jumbo Critical Point Dryer 13200J AB, a yield of 116.5 mg (77.5%) was quantified. The structure could be determined using SC‐XRD with additional Rietveld refinement from PXRD data. TGA (rest mass): 23.47% ZnO. IR υ cm^−1^ = 2952 (w), 2926 (w), 2856 (w), 1728 (w), 1604 (m), 1556 (m), 1551 (m), 1494 (w), 1471 (w), 1430 (m), 1390 (s), 1286 (s), 1230 (w), 1205 (w), 1168 (w), 1118 (m), 1088 (w), 1037 (w), 987 (w), 958 (w), 920 (w), 879 (w), 860 (w), 834 (w), 780 (m), 738 (w), 717 (w), 689 (w), 670 (w), 659 (w), 621 (w), 608 (w).

##### Molecular Modeling

*DFT Geometry Optimization*: Experimentally synthesized DUT‐193 crystal structure was geometry optimized using periodic density‐functional theory (DFT) calculations as implemented in the quickstep module^[^ ^44^ ^]^ of the CP2K program^[^ ^45^ ^]^ with the Gaussian plane wave protocol. The general gradient approximation to the exchange–correlation functional according to Perdew–Burke–Ernzerhof^[^ ^46^ ^]^ was used in combination with Grimme′s DFT‐D3 semiempirical dispersion corrections.^[^ ^47^ ^]^ The optimized triple‐ζ plus valence‐polarized Gaussian‐type basis sets (TZVP‐MOLOPT) were considered for all atoms, except for the Zn metal centers, for which shorter‐range double‐ζ plus valence polarization functions (DZVP‐MOLOPT) were employed.^[^ ^48^ ^]^ The interactions between core electrons and valence shells of the atoms were defined by the pseudopotentials derived by Goedecker, Teter, and Hutter.^[^ ^49^ ^]^ The auxiliary plane wave basis sets were truncated at 500 Ry. Atomic partial charges of DUT‐193 framework atoms were further derived by applying the REPEAT fitting strategy^[^ ^50^ ^]^ for the periodic system as implemented in the CP2K code.

##### Structure Textural Properties

Textural properties of the DUT‐193 structure were calculated using Zeo++,^[^ ^27^ ^]^ which includes the largest cavity diameter of 29.4 Å, the pore limiting diameter of 6.3 Å, PV of 2.1 cm^3^ g^−1^, void fraction (*ϕ*) of 0.83, and N_2_‐accessible SA of 4480 m^2^ g^−1^. For SA calculations, the trial number was set to 5000 and the kinetic radius of N_2_ used was 1.84 Å. For PV calculations, the trial number was set to 50 000, and the probe radius used was 0 Å.

##### Monte Carlo Simulations

GCMC simulations were performed to predict the single‐component adsorption isotherms of Ar (87 K), N_2_ (77 K), CH_4_ (91 K and 111 K), and CO_2_ (195 K) in DUT‐193 with pressure ranging from 10^−6^ to 1 bar. All the GCMC simulations were achieved using the Complex Adsorption and Diffusion Simulation Suite. A simulation box consisting of a single unit cell (1 × 1 × 1) was considered for DUT‐193, and the atomic positions of the MOF frameworks were held fixed during the adsorption and trial moves for the guest molecules, including, i.e., translational, rotational, creation, and deletion. The host–guest nonbonded Lennard‐Jones (LJ) interactions were computed in real space using a cutoff of 12.0 Å, while the interactions between unlike force field centers were treated by means of the Lorentz–Berthelot combination rule. The LJ parameters for the MOF framework atoms were described using the universal force field (Table S1, Supporting Information).^[^ ^51^ ^]^ Argon^[^ ^52^ ^]^ was represented by a single LJ site described by the optimized potentials for liquid simulations force field, and the other guest molecules CH_4_,^[^ ^53^ ^]^ N_2_,^[^ ^54^ ^]^ and CO_2_^[^ ^54^ ^]^ parameters were taken from the transferable potentials for phase equilibria (TraPPE) parameters (Table S2, Supporting Information). In the case of N_2_ and CO_2_ adsorption, the long‐range electrostatic interactions were calculated using the Ewald summation technique with an accuracy of 1 × 10^−6^. For each pressure point, 2 × 10^8^ Monte Carlo production steps were considered. The Peng–Robinson equation of state was used to determine the gas‐phase fugacity for all the guest molecules.^[^ ^55^ ^]^

## Conflict of Interest

The authors declare no conflict of interest.

## Supporting information

Supplementary Material

## Data Availability

The data that support the findings of this study are available in the supplementary material of this article.
