# Supplementary material for: Nanoporous Frameworks with High Porosity and Unexpected Rigid Framework Topologies Based on Odd‐Numbered Ring‐Expanded Linkers
Source: Small Sci. 2023 Nov 20;3(12):2300158. doi: 10.1002/smsc.202300158 (PMC11935872; doi:10.1002/smsc.202300158)
Supplement: Supplementary file 1 — Supplementary Material [file SMSC-3-2300158-s001.pdf]

WILEY-VCH

Supporting Information

Nanoporous Frameworks with High Porosity and Unexpected Rigid Framework Topologies Based on Odd-Numbered Ring-Expanded Linkers

*Bodo Felsner[a], Karuppasamy Gopalsamy[b], Volodymyr Bon[a], Irena Senkovska[a], Guillaume Maurin[b], Stefan Kaskel[a]**

Table of contents

1. Linker synthesis and characterization 2
1.1 Synthesis scheme 2
1.2 NMR spectra 3
1.3 ATR-IR spectra 10
2. MOF Characterization 11
2.1 DUT-184 11
2.2 DUT-193 13
2.3 DUT-193 Grand Canonical Monte Carlo (GCMC) Simulations 18

1. Linker synthesis and characterization

1.1 Synthesis scheme

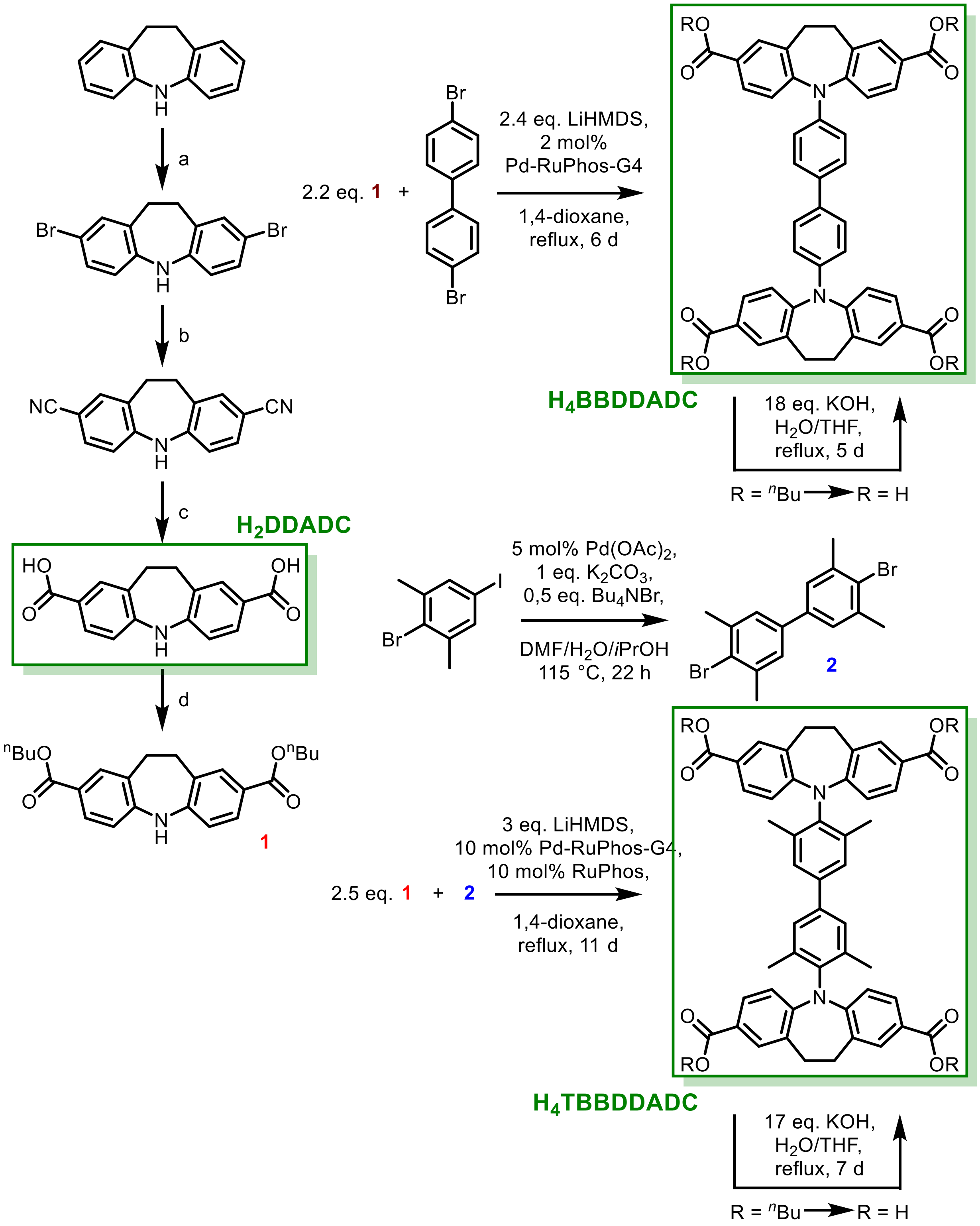

ESI Fig. S1: Synthesis scheme of all linkers consisting of the 4 step synthesis of the precursor Bu_2DDADC, the one-step synthesis of the second linker bridge and the following Buchwald-Hartwig couplings and hydrolyses towards $H_4BBDDADC$ and $H_4TBBDDADC$. a) Bromination: NBS, SiO_2, DCM, 25 °C, 2.5 h, b) Cyanation: $Zn(CN)_2$, $Zn(OAc)_2$, Zn, $Pd_2(dba)_3$, dppf, DMF, 80 °C, 8 d, c) Nitrile hydrolysis: 1. NaOH, CuI, H_2O, reflux, 7 d, 2. HCl, d) Esterification: $^{n}BuOH$, H_2SO_4, 120 °C, 4 d.

1.2 NMR spectra

a)

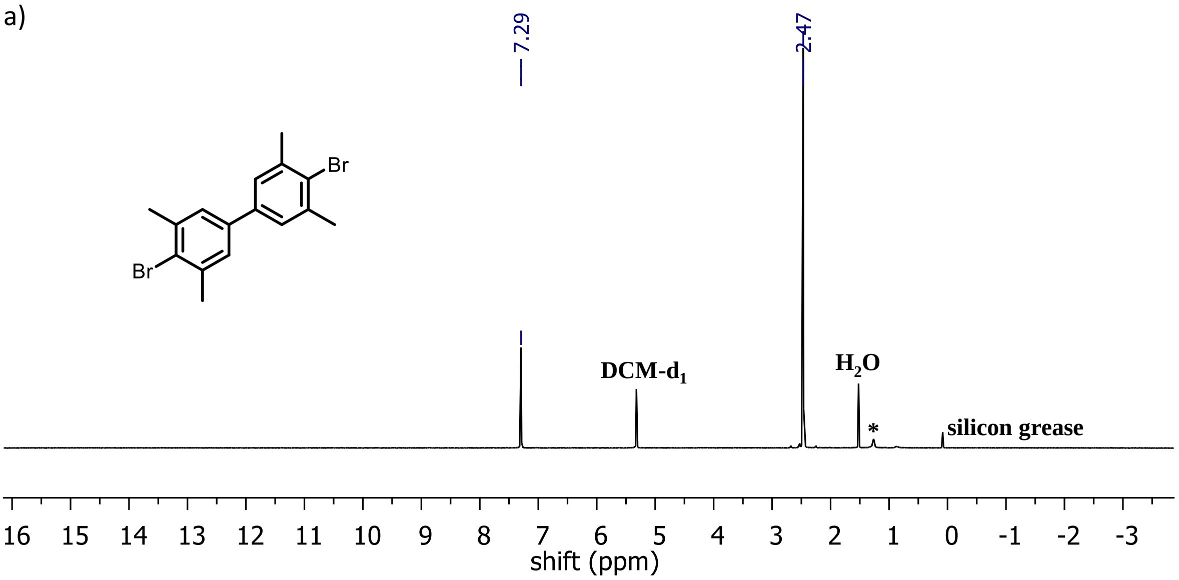

b)

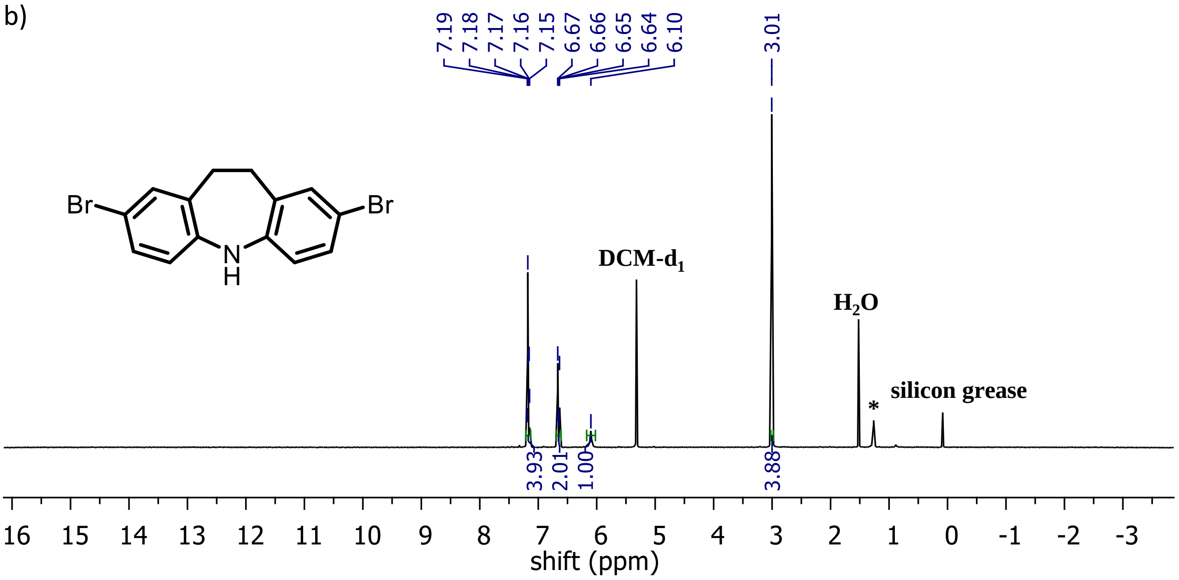

ESI Fig. S2: ^{1}H NMR of a) 4,4'-dibromo-3,3',5,5'-tetramethyl-1,1'-biphenyl and b) 2,8-dibromo-10,11-dihydro-5*H*-dibenzo[*b*,*f*]azepine in DCM-d_2; * unknown impurity.

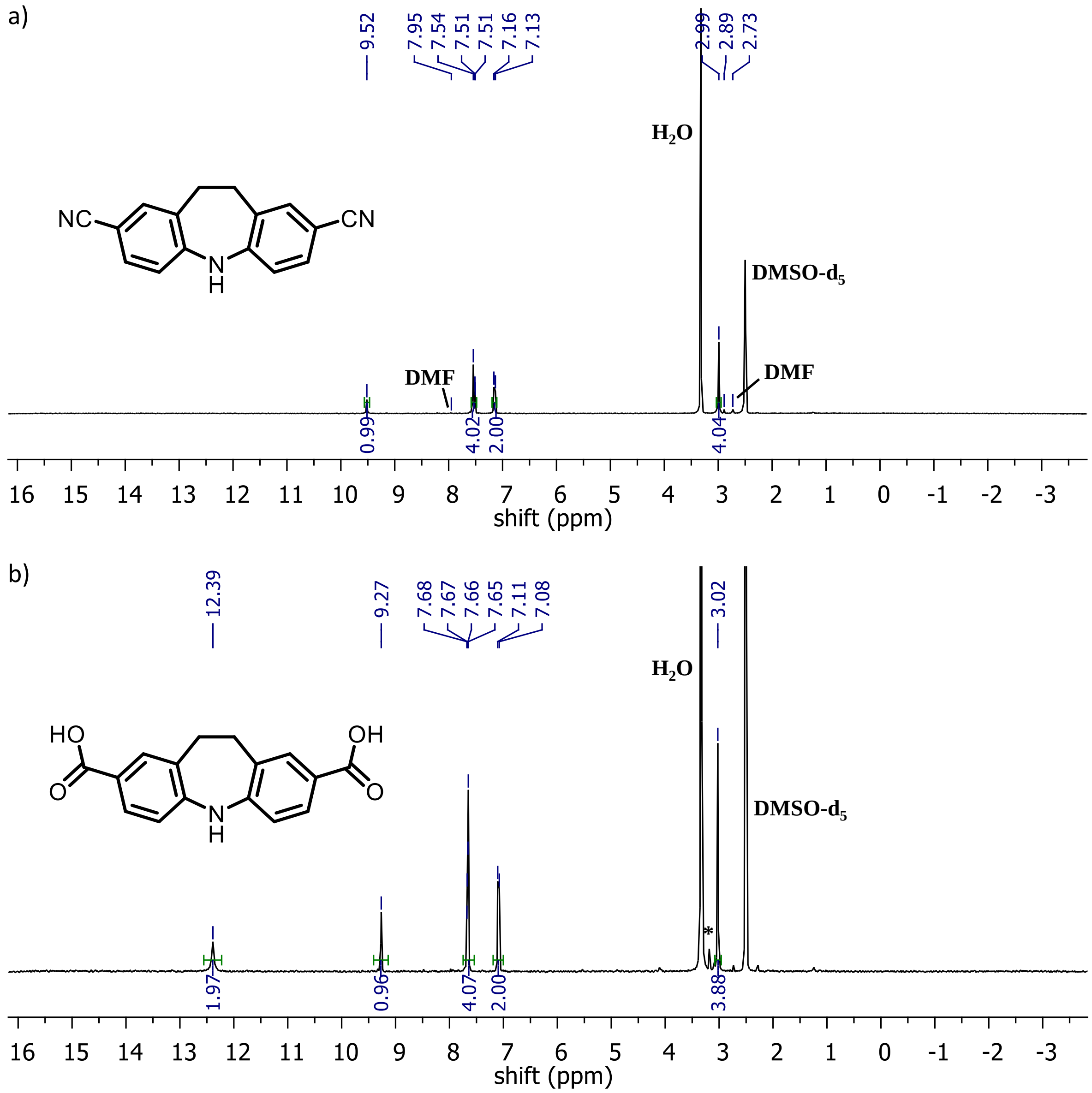

ESI Fig. S3: ^{1}H NMRs of a) 10,11-dihydro-5*H*-dibenzo[*b*,*f*]azepine-2,8-dicarbonitrile and b) 10,11-dihydro-5*H*-dibenzo[*b*,*f*]azepine-2,8-dicarboxylic acid (H_2DDADC) in DMSO-d_6; * unknown impurity.

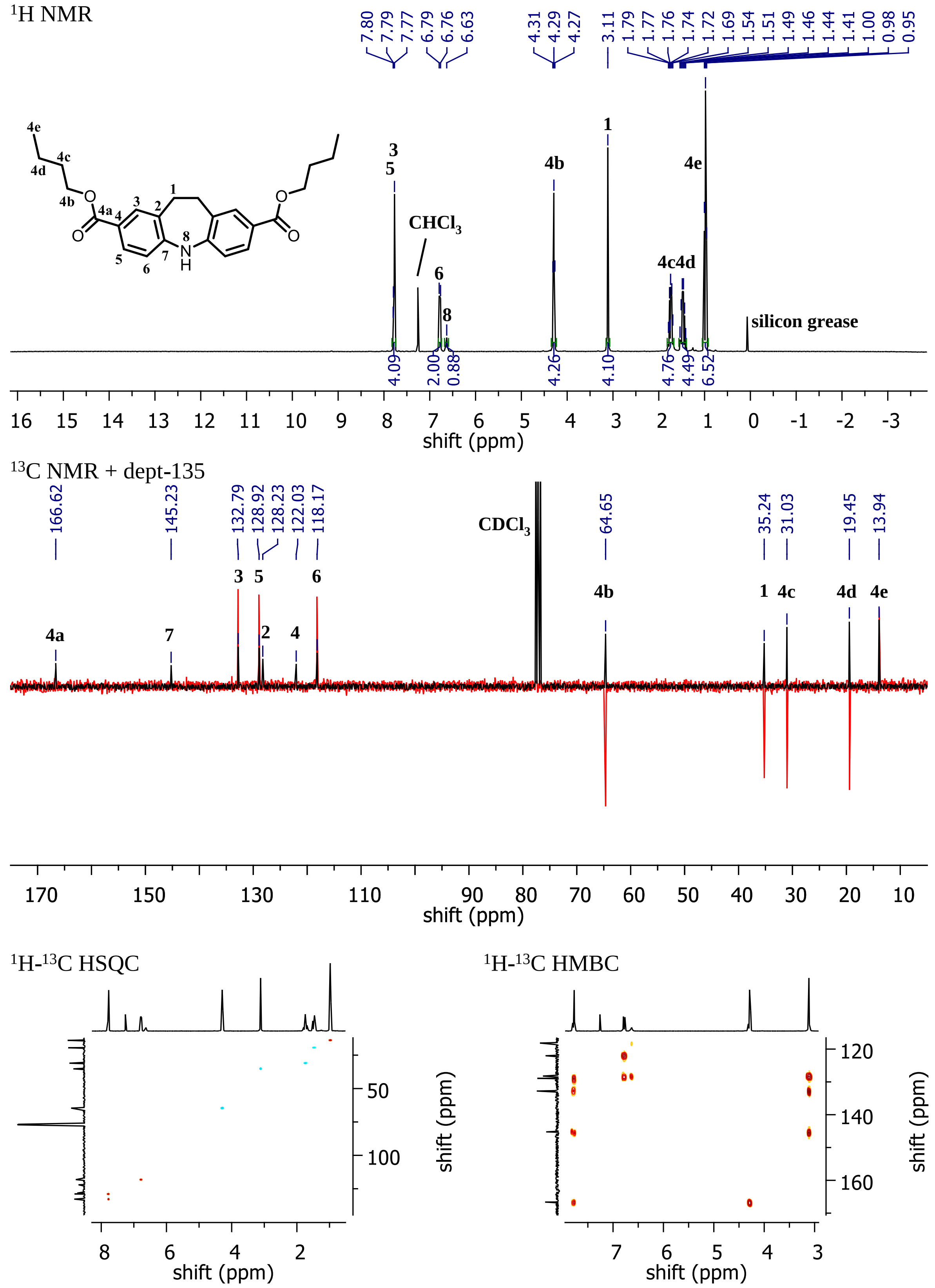

ESI Fig. S4: Sections of the NMR spectra of dibutyl 10,11-dihydro-5*H*-dibenzo[*b*,*f*]azepine-2,8-dicarboxylate ($^{n}Bu_2$DDADC) in $CDCl_3$. Top: ^{1}H NMR (300 MHz), central: ^{13}C NMR (75 MHz) (black) and dept-135 (red), bottom left: ^{1}H-^{13}C HSQC, bottom right: ^{1}H-^{13}C HMBC.

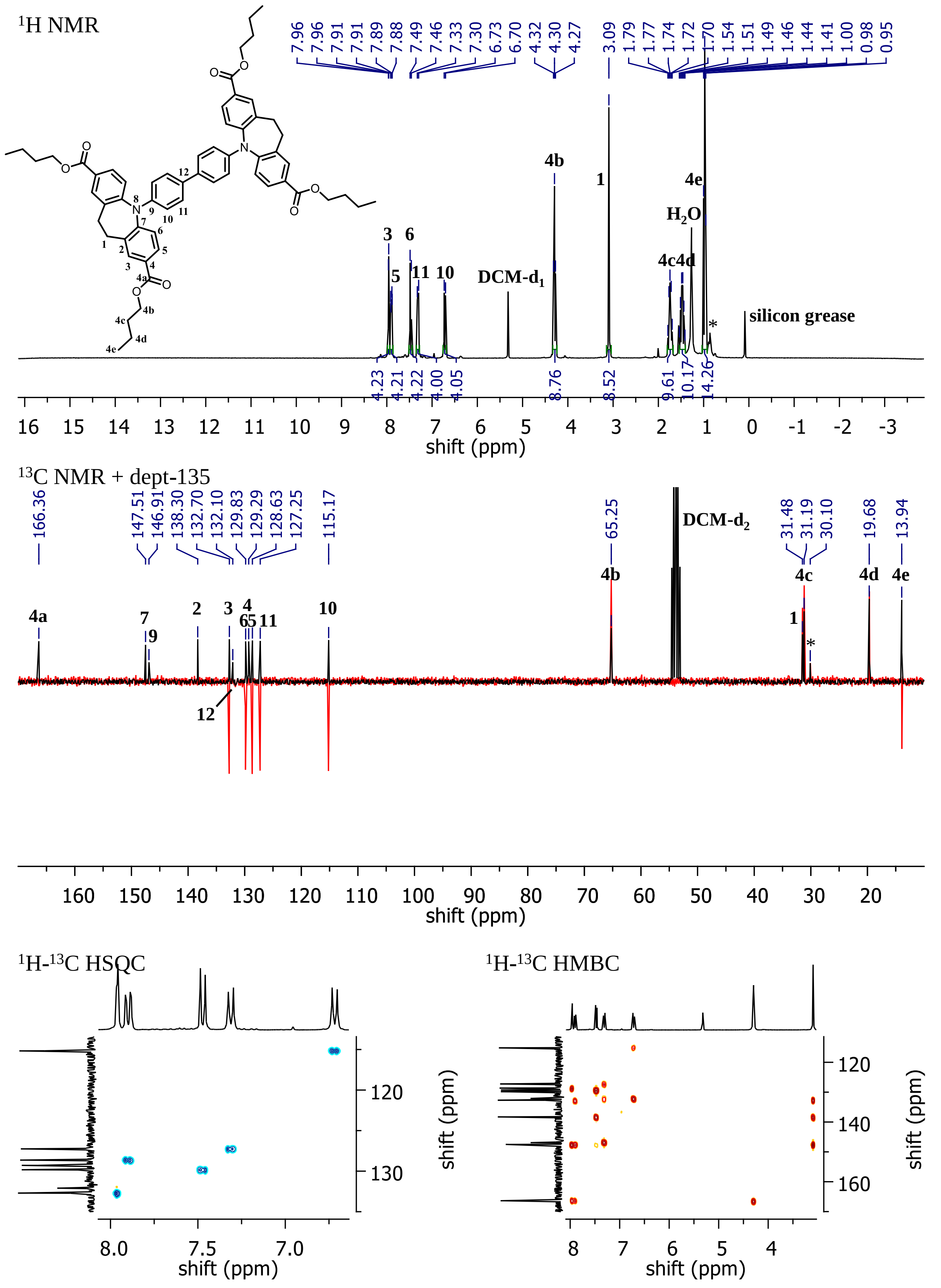

ESI Fig. S5: Sections of the NMR spectra of tetrabutyl 5,5'-([1,1'-biphenyl]-4,4'-diyl)bis(10,11-dihydro-5*H*-dibenzo[*b*,*f*]azepine-2,8-dicarboxylate) $^{n}Bu_4$BBDDADC in DCM-d_2. Top: ^{1}H NMR (300 MHz), central: ^{13}C NMR (75 MHz) (black) and dept-135 (red), bottom left: ^{1}H-^{13}C-HSQC, bottom right: ^{1}H-^{13}C-HMBC; * unknown impurities.

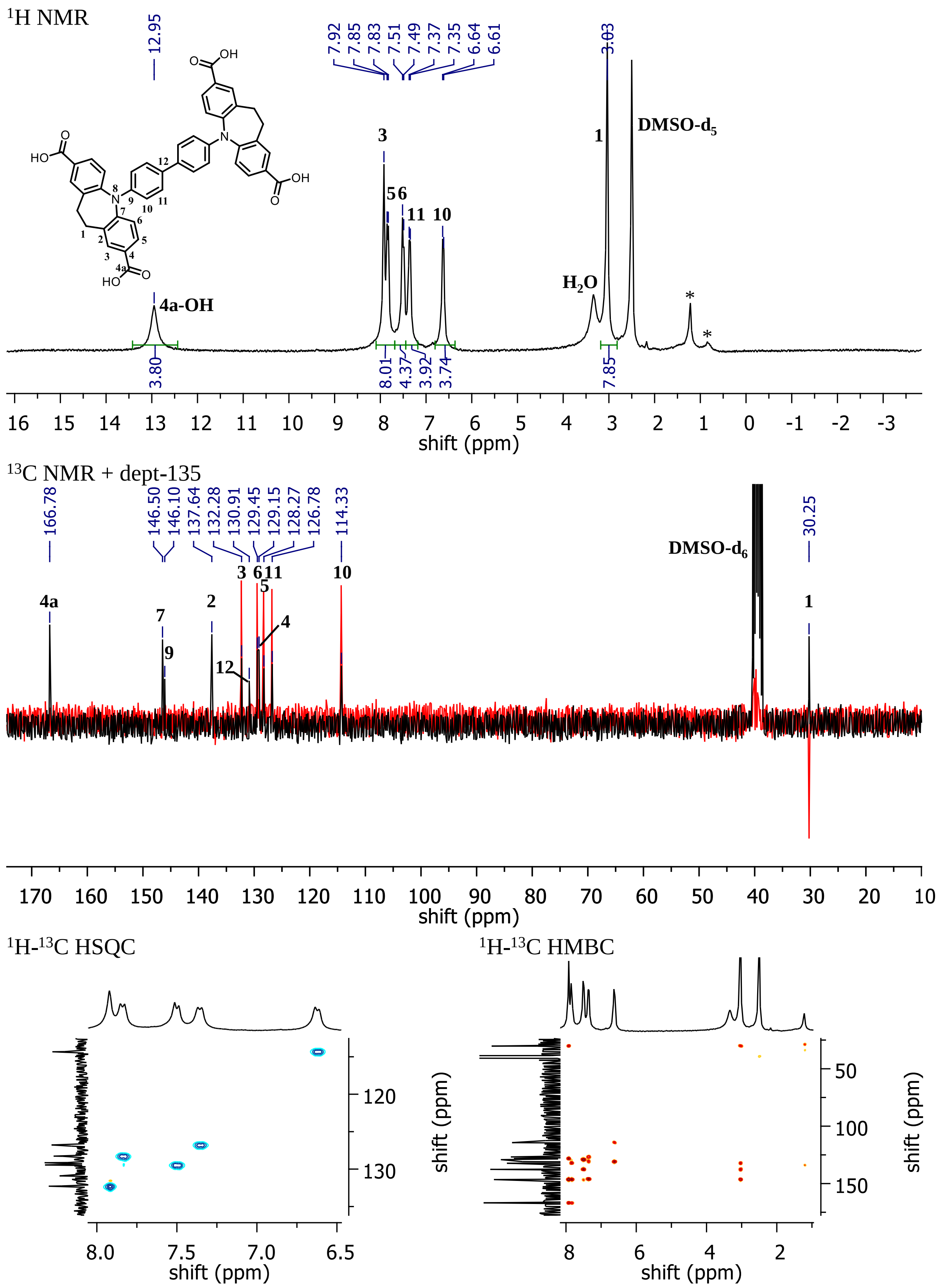

ESI Fig. S6: Sections of the NMR spectra of 5,5'-([1,1'-biphenyl]-4,4'-diyl)bis(10,11-dihydro-5*H*-dibenzo[*b*,*f*]azepine-2,8-dicarboxylic acid) H_4BBDDADC in DMSO-d_6. Top: ^{1}H NMR (300 MHz), central: ^{13}C NMR (75 MHz) (black) and dept-135 (red), bottom left: ^{1}H-^{13}C-HSQC, bottom right: ^{1}H-^{13}C-HMBC; * unknown impurities.

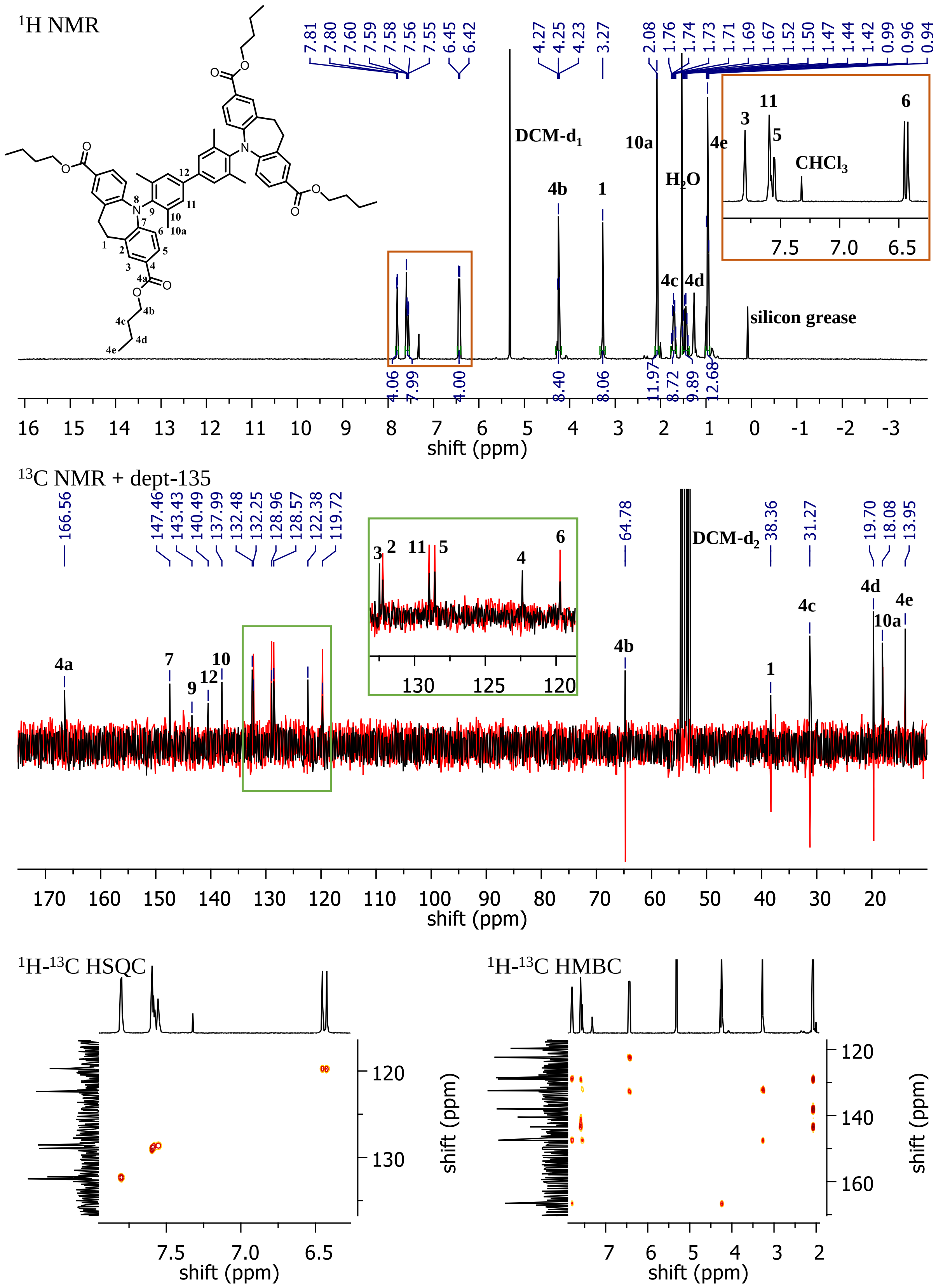

ESI Fig. S7: Sections of the NMR spectra of tetrabutyl 5,5'-(3,3',5,5'-tetramethyl-[1,1'-biphenyl]-4,4'-diyl)bis(10,11-dihydro-5*H*-dibenzo[*b*,*f*]azepine-2,8-dicarboxylate) ^{n}Bu$_4$TBBDDADC in DCM-d$_2$. Top: ^{1}H NMR (300 MHz), central: ^{13}C NMR (75 MHz) (black) and dept-135 (red), bottom left: ^{1}H-^{13}C-HSQC, bottom right: ^{1}H-^{13}C-HMBC.

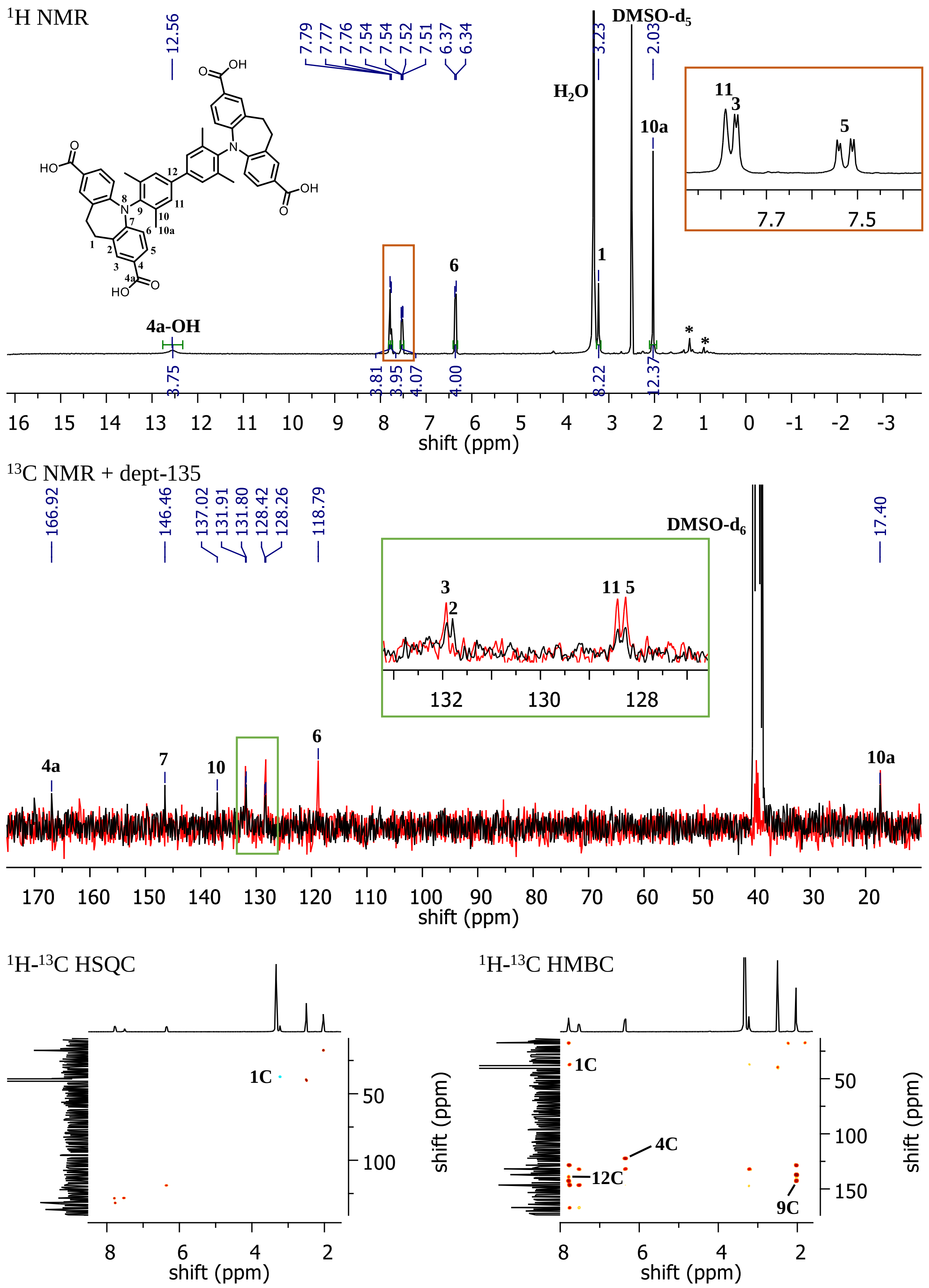

ESI Fig. S8: Sections of the NMR spectra of 5,5'-(3,3',5,5'-tetramethyl-[1,1'-biphenyl]-4,4'-diyl)bis(10,11-dihydro-5*H*-dibenzo[*b*,*f*]azepine-2,8-dicarboxylic acid) H_4TBBDDADC in DMSO-d_6. Top: ^{1}H NMR (300 MHz), central: ^{13}C NMR (75 MHz) (black) and dept-135 (red), bottom left: ^{1}H-^{13}C-HSQC, bottom right: ^{1}H-^{13}C-HMBC. Some ^{13}C signals could only be found in HSQC and HMBC experiments and are therefore assigned there.

1.3 ATR-IR spectra

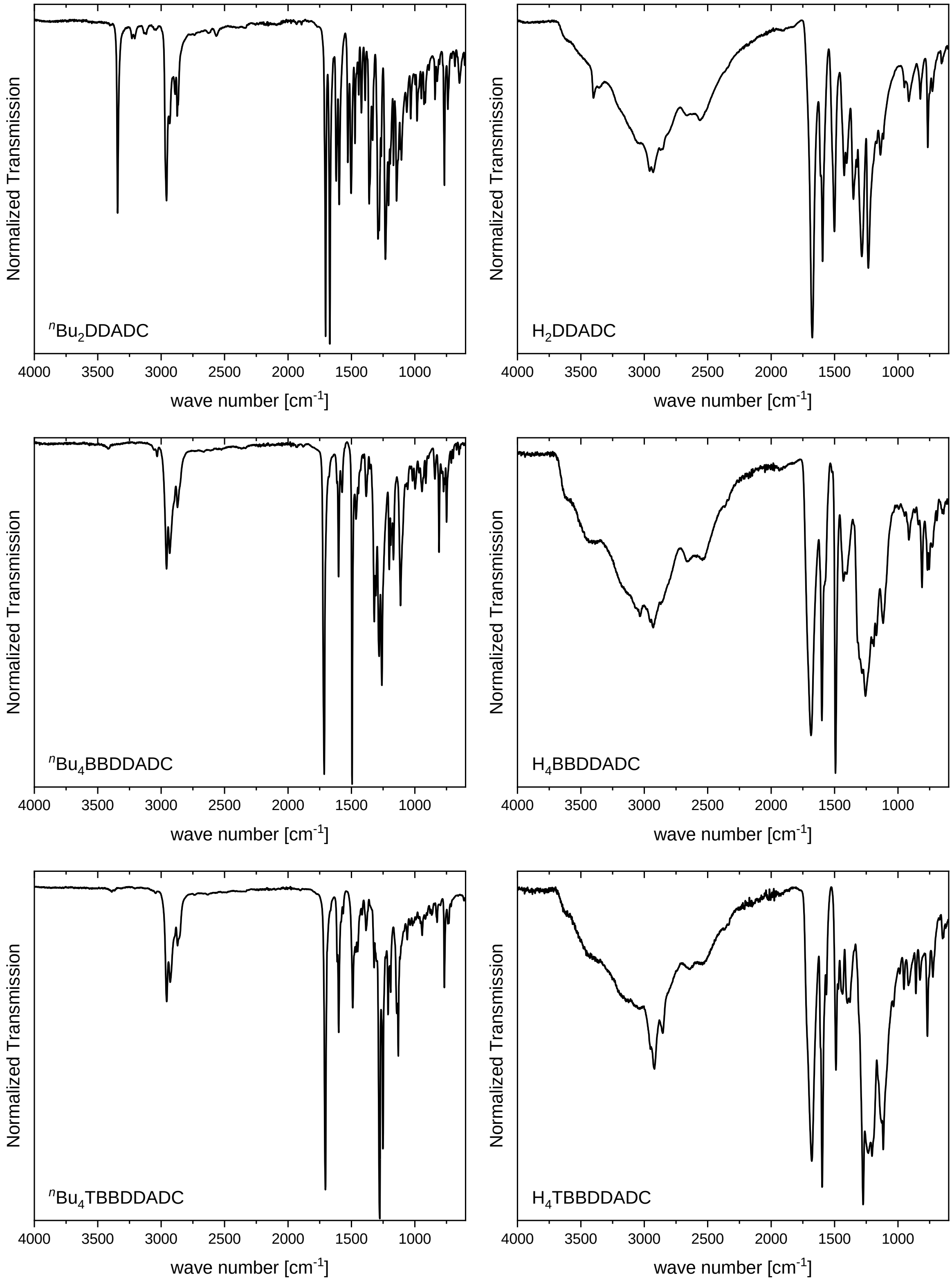

ESI Fig. S9: ATR-IR spectra of all linkers and their corresponding *n*-butyl esters.

2. MOF Characterization

2.1 DUT-184

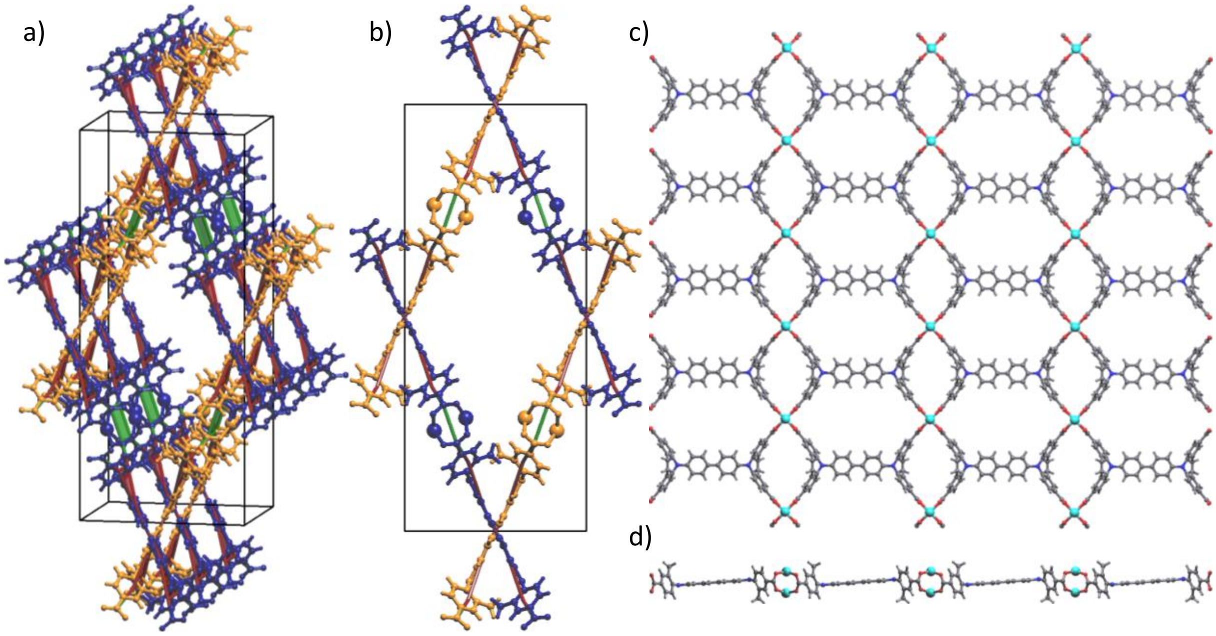

ESI Fig. S10: Various views on the structure and underlying augmented net of DUT-184 (NMP molecules omitted) with highlighted layer sets (orange and dark blue) in a) and b) and a single layer viewed from top (c) and side (d). Representation of atoms: carbon – grey, nitrogen – blue, oxygen – red, zinc – light blue, hydrogen – light grey.

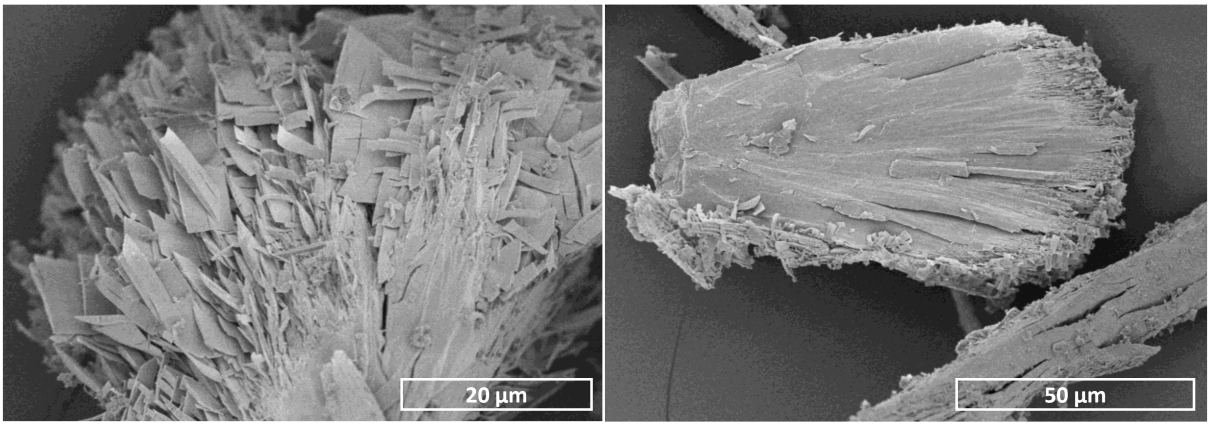

ESI Fig. S11: SEM images of DUT-184.

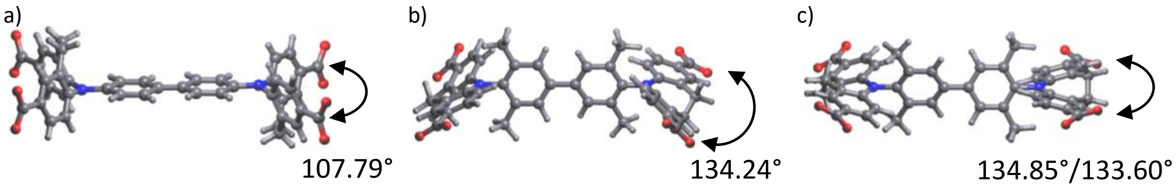

ESI Fig. S12: Comparison of linkers and their angles between carboxylates in the DUT-184 (a) and DUT-193 (b and c) structures.

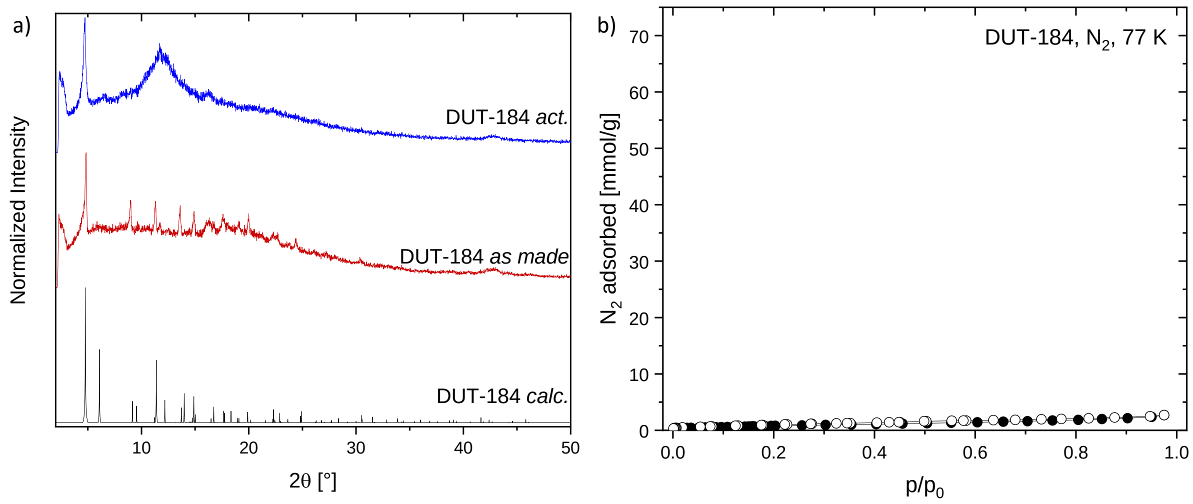

ESI Fig. S13: a) PXRD patterns of DUT-184 calculated from the crystal structure (black), as made (red) and activated (blue); b) physisorption of nitrogen at 77 K on DUT-184.

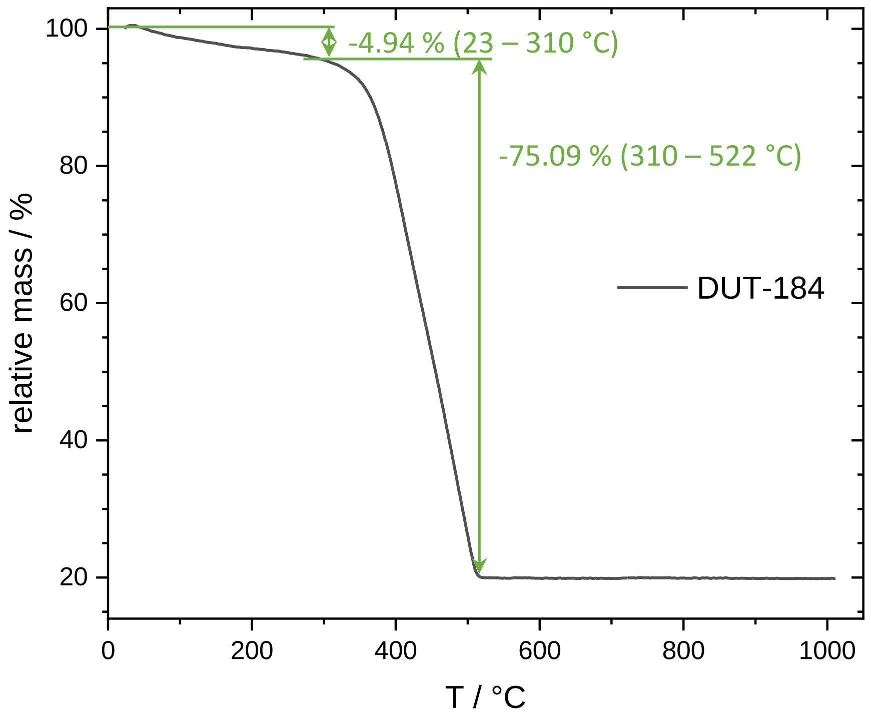

ESI Fig. S14: TGA of DUT-184 in air with a heating rate of 5 K/min.

2.2 DUT-193

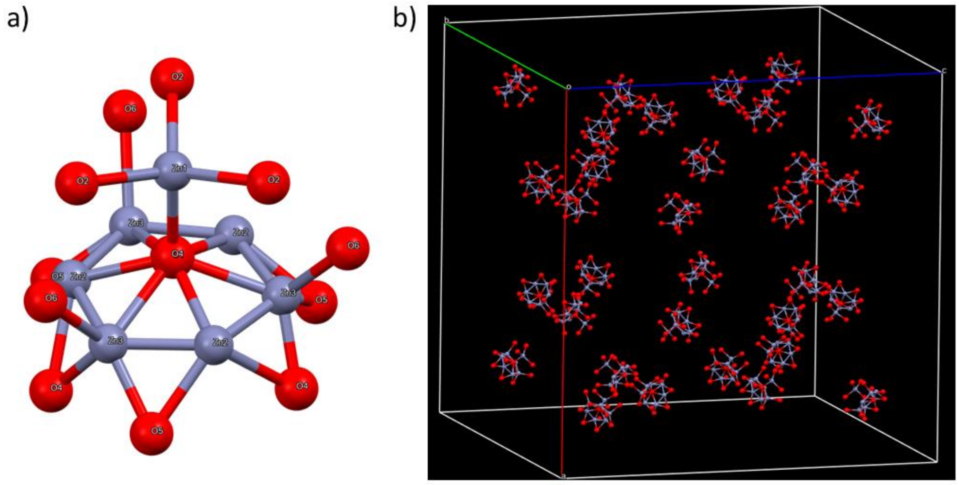

ESI Fig. S15: Disordered structure of DUT-193, derived from single crystal X-ray diffraction experiment: a) disordered Zn_4O cluster; b) packing of the clusters in the unit cell.

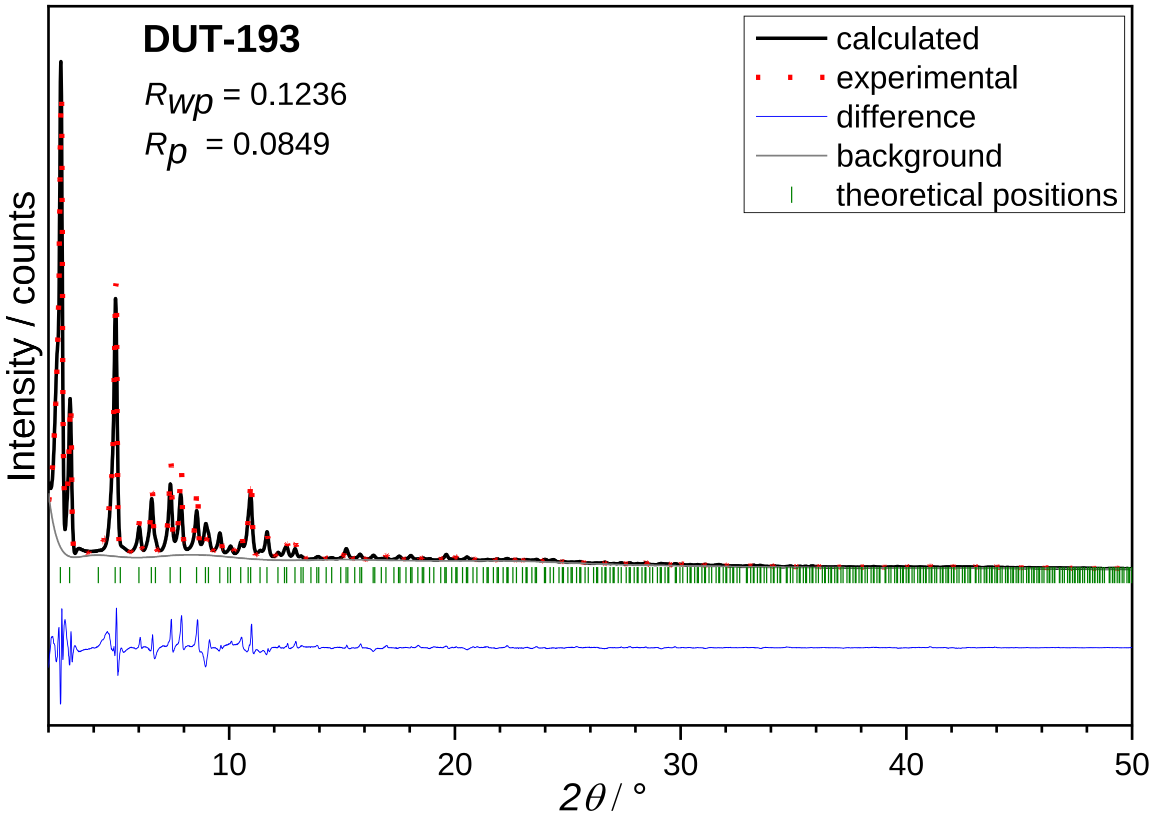

ESI Fig. S16: Rietveld plot for DUT-193.

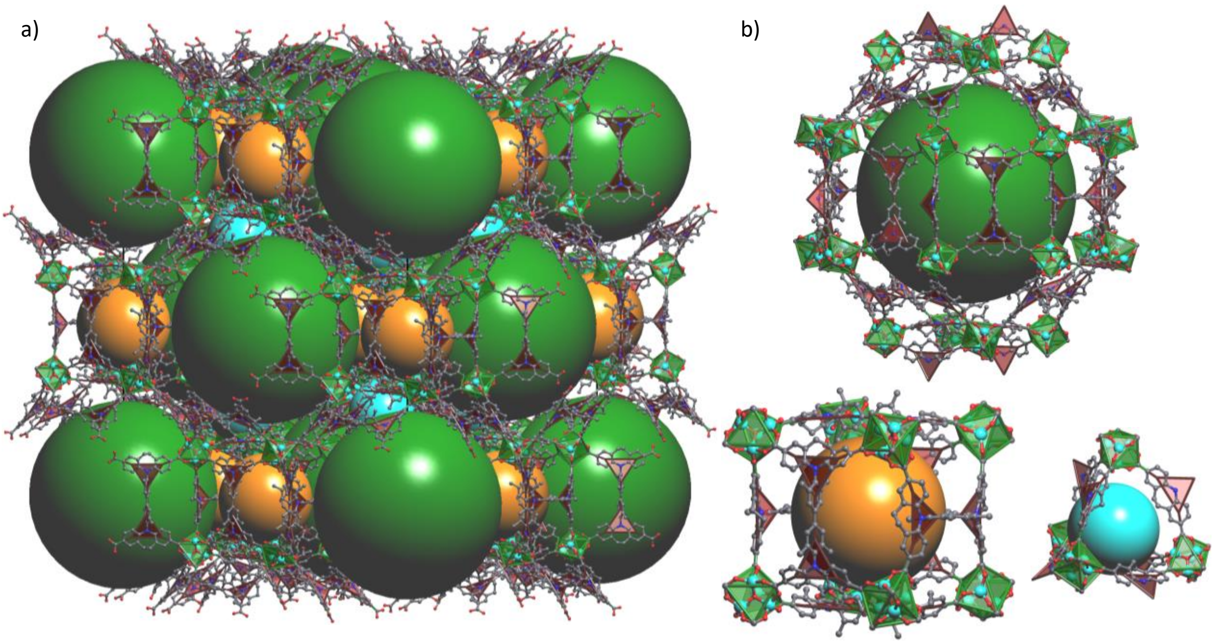

ESI Fig. S17: View on the DUT-193 pore structures: a) packaging in the unit cell; b) each pore separately. Atoms: carbon – grey, nitrogen – blue, oxygen – red, zinc – light blue, hydrogen –omitted for better overview. Topological representation: green octahedra – simplified $Zn_4O(CO_2)_6$ cluster, two connected red triangles – simplified TBBDDADC linker. Pores: rhombicuboctahedral mesopore – green, cubic micropore – orange, tetrahedral micropore – light blue.

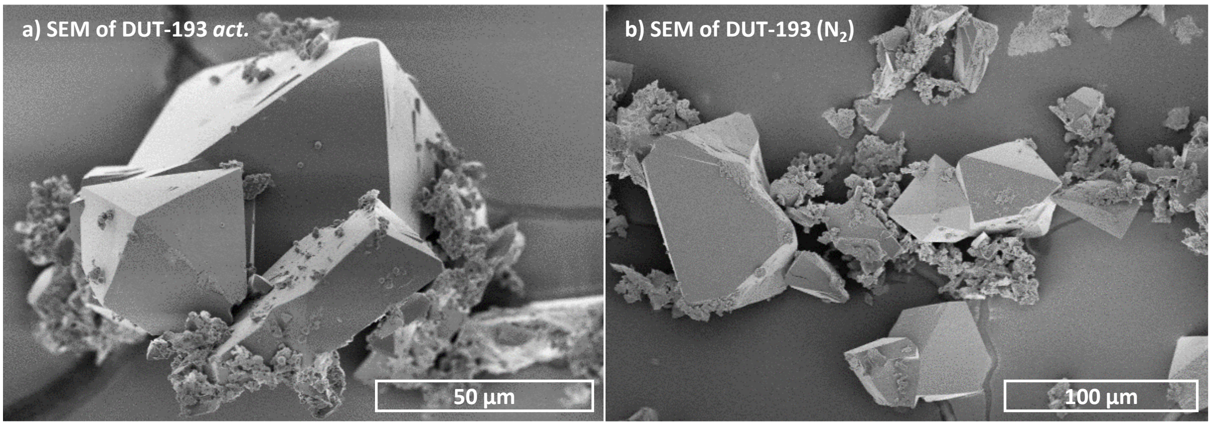

ESI Fig. S18: a) SEM image of the activated DUT-193 sample; b) SEM image of DUT-193 after N_2 physisorption.

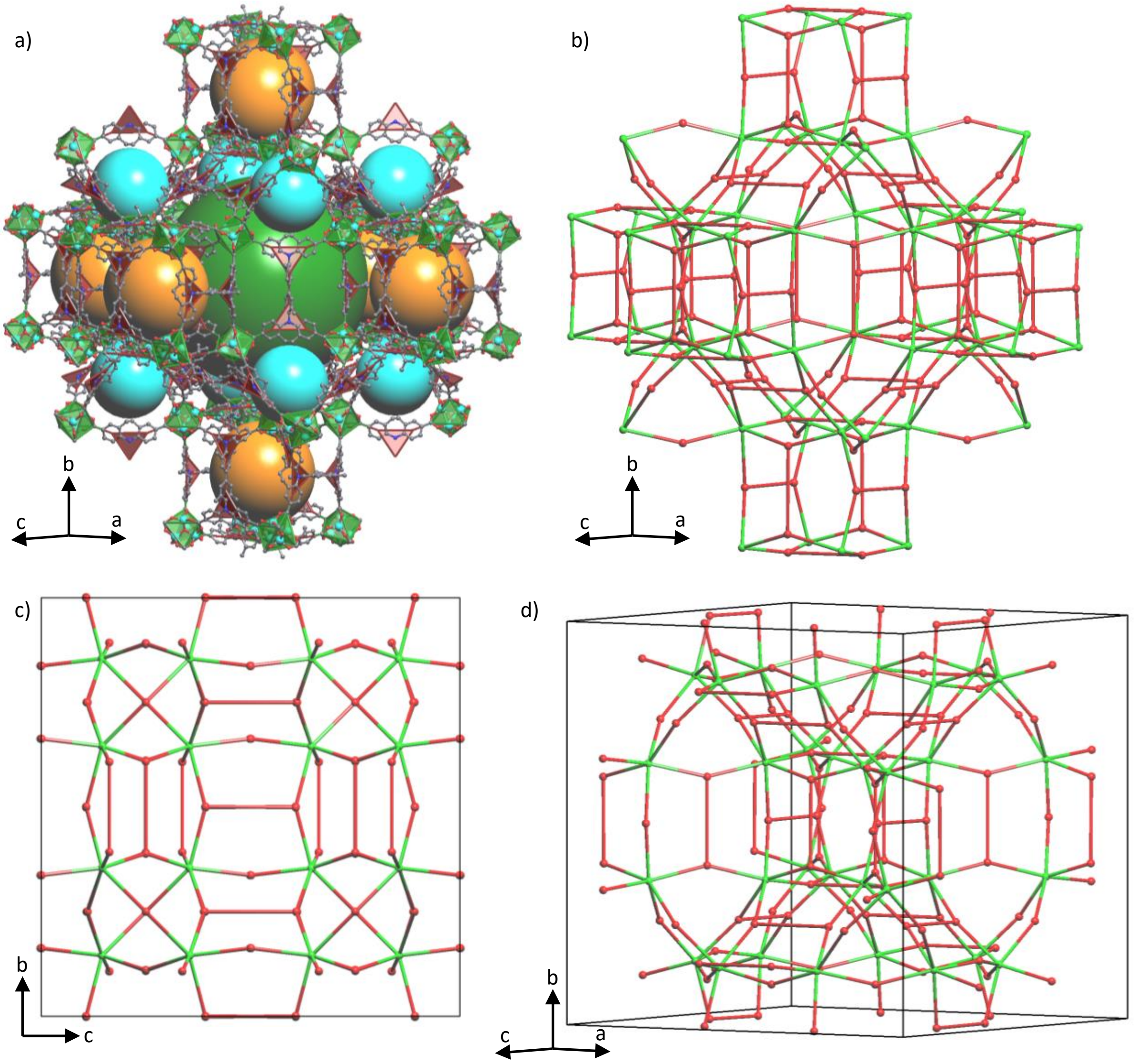

ESI Fig. S19: View on the DUT-193 structure, topology and net: a) all pores around the mesopore, b) net of all pores around the mesopore, c) net in 1 0 0 perspective and d) net in 10 1 15 perspective. Nodes: green: $Zn_4O(CO_2)_6$ cluster, red: TBBDDADC linker split into two 3-c nodes.

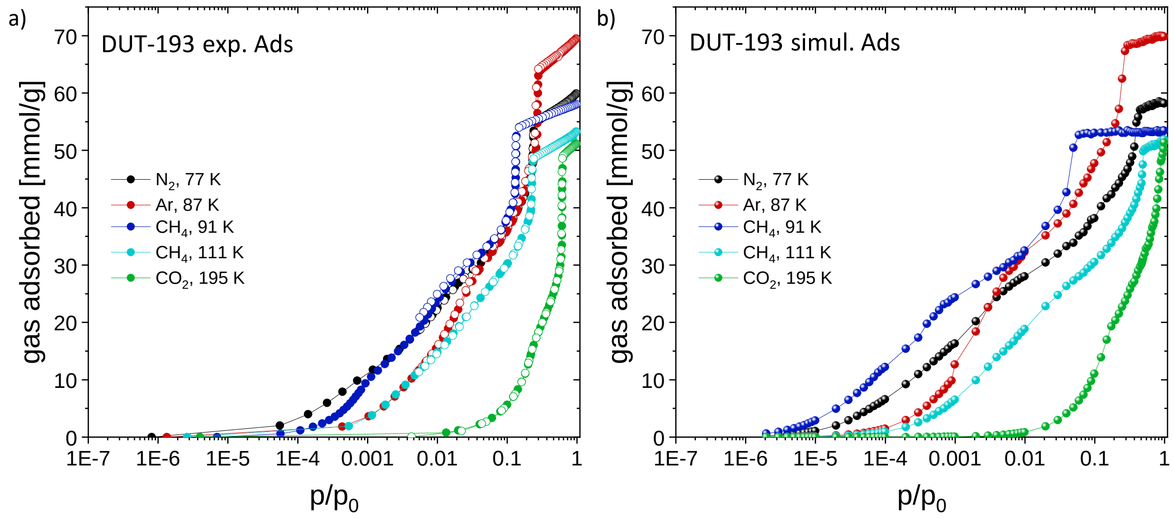

ESI Fig. S20: A semi-logarithmic plot of the adsorption isotherms of various gases on DUT-193: a) experiment (adsorption: filled symbols, desorption: empty symbols) and b) simulation (only adsorption) at different temperatures as stated in the legend.

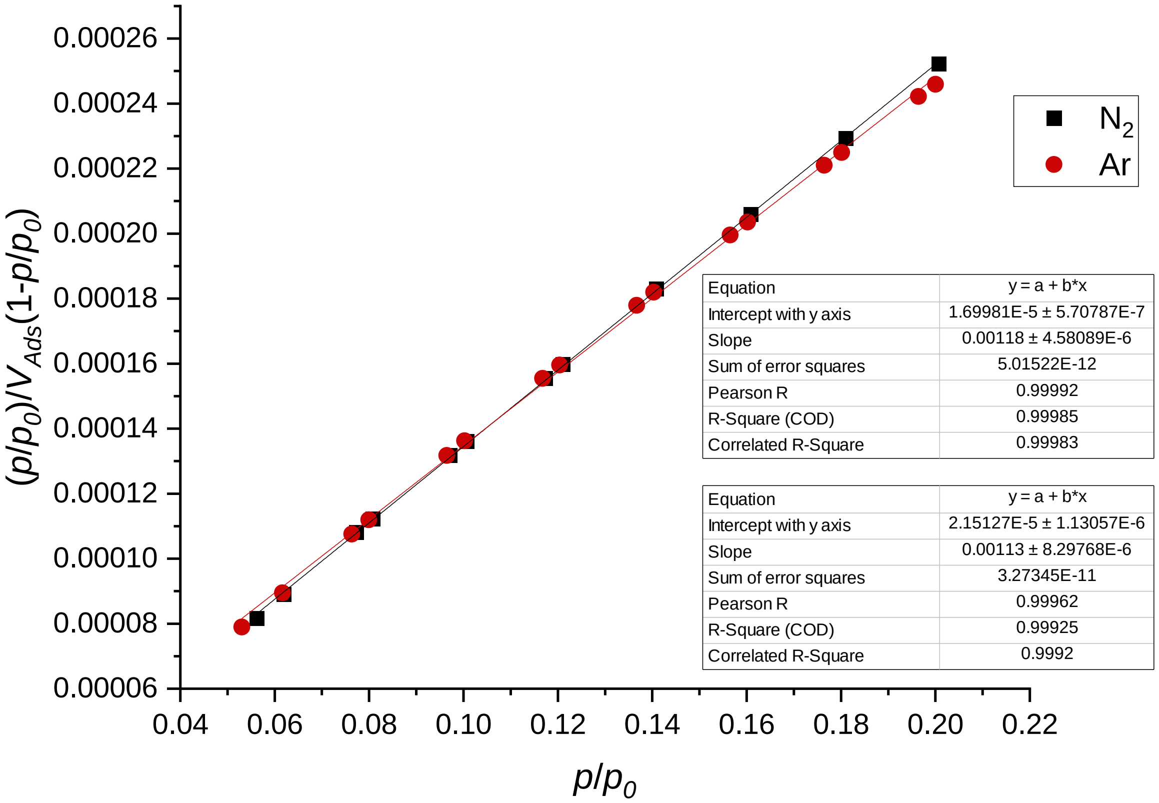

ESI Fig. S21: BET plot based on nitrogen (black) and argon (red) physisorption on DUT-193 at their corresponding boiling temperatures in the relative pressure range between 0.05 to 0.21. Resulting specific surface areas: nitrogen: 3288 m^2/g (C = 70), argon: 2911 m^2/g (C = 54).

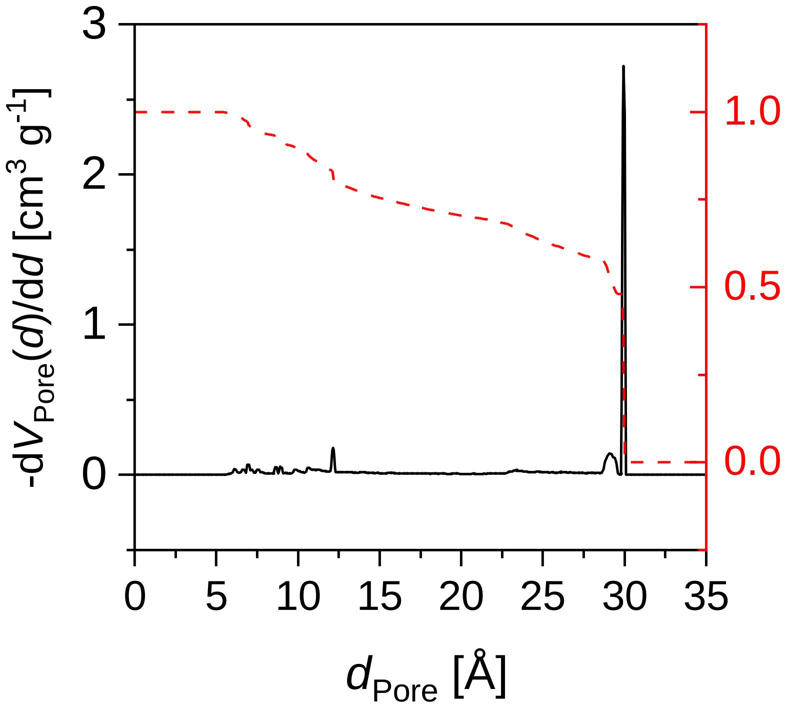

ESI Fig. S22: Pore size distribution of DUT-193.

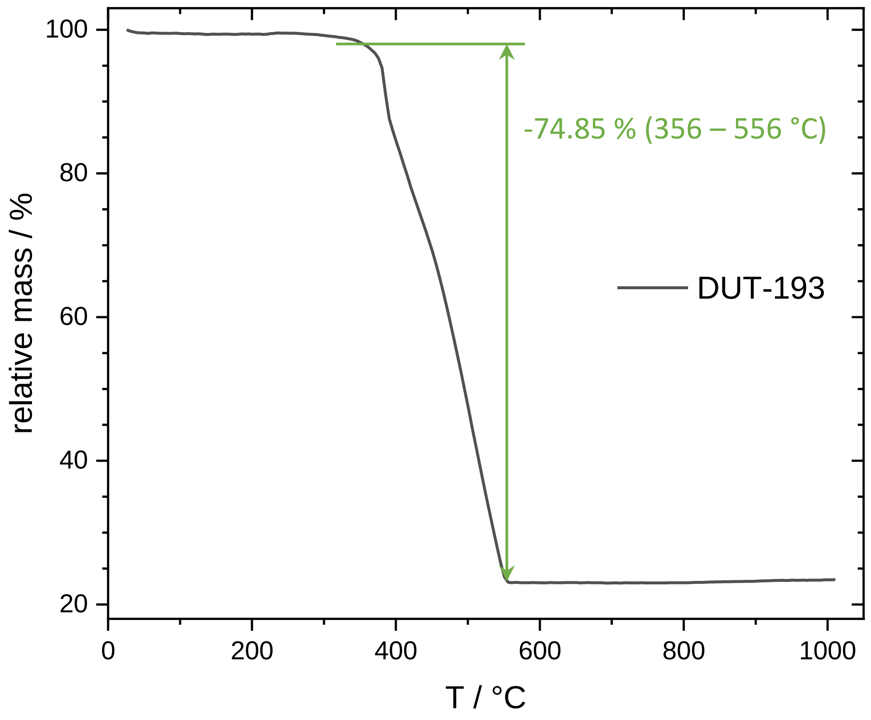

ESI Fig. S23: TGA of DUT-193 in air with a heating rate of 5 K/min.

2.3 DUT-193 Grand Canonical Monte Carlo (GCMC) Simulations

Table S1: Lenard-Jones (LJ) potential parameters for the atoms in DUT-193, taken from universal force field (UFF).[1]

MOF Atoms	σ (Å)	ε/kB (K)
C	3.43	52.83
H	2.57	22.14
N	3.26	34.74
O	3.12	30.19
Zn	2.46	62.44

Table S2: LJ potential parameters and electrostatic charges for the CH_4, N_2 and CO_2 molecules, taken from Transferable Potentials for Phase Equilibria (TraPPE).[2]

Molecules	Atom	σ (Å)	ε/kB (K)	q
CH_4	CH_4_UA	3.73	148.0	-
N_2	N2_N	3.31	36.0	-0.482
	N2_N_{com}	0.0	0.0	0.964
CO_2	C_CO_2	2.80	27.0	0.700
	O_CO_2	3.05	79.0	-0.350

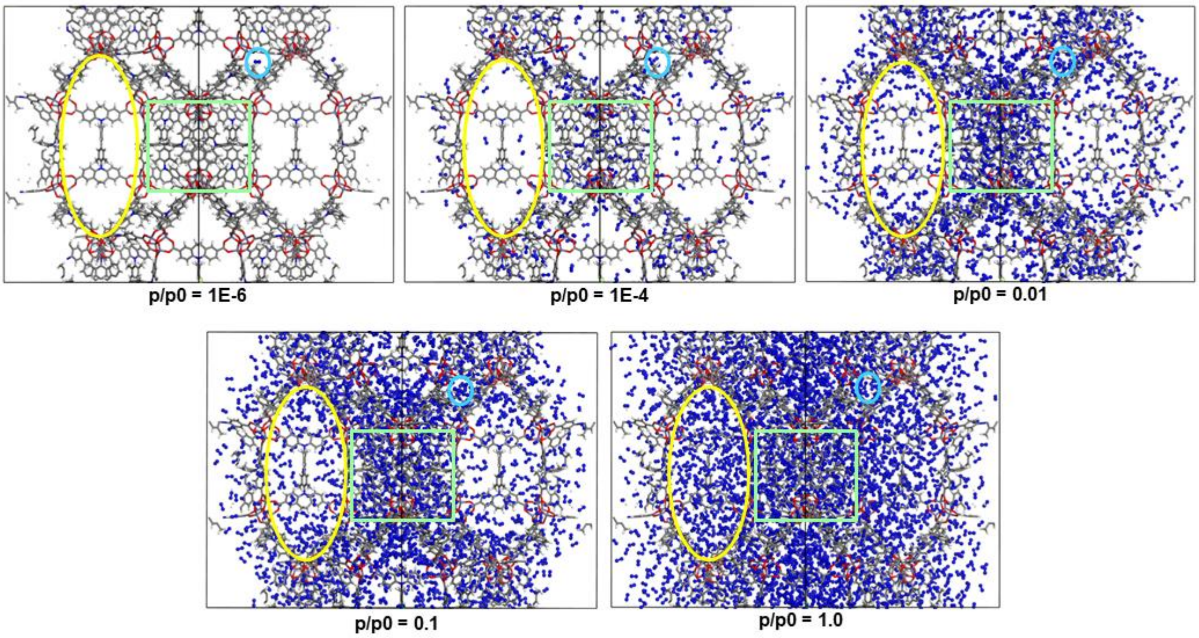

ESI Fig. S24: Illustrative GCMC configurations of N_2 adsorption in DUT-193 at 77 K with p/p_0 ranging from 10^{-6} to 1 bar.

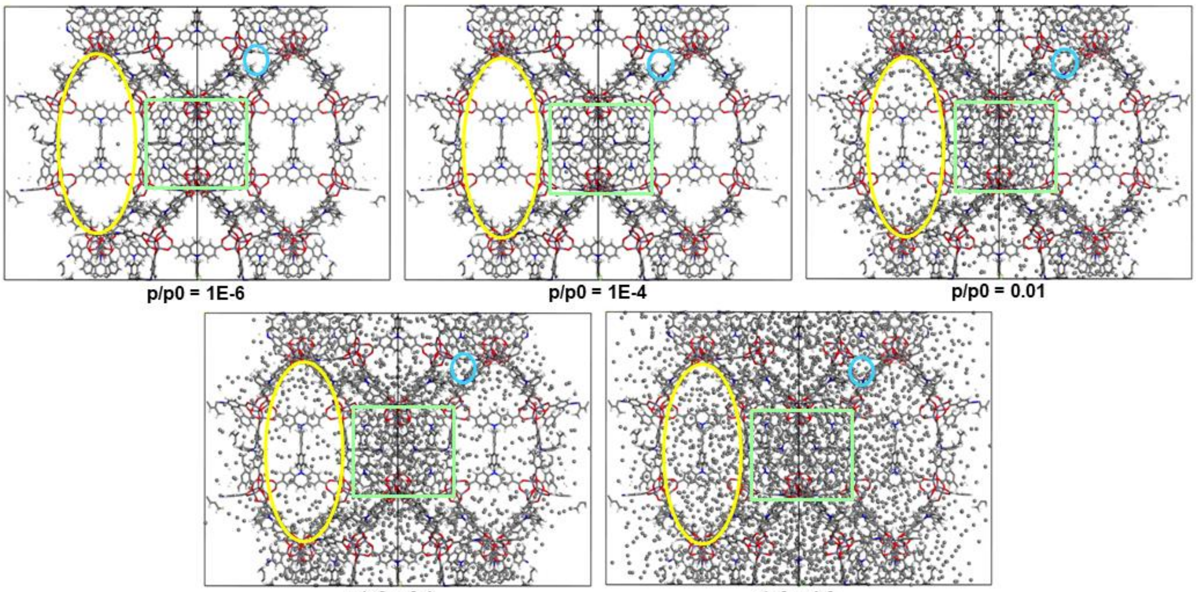

ESI Fig. S25: Illustrative GCMC configurations of CH_4 adsorption in DUT-193 at 77 K with p/p_0 ranging from 10^{-6} to 1 bar.

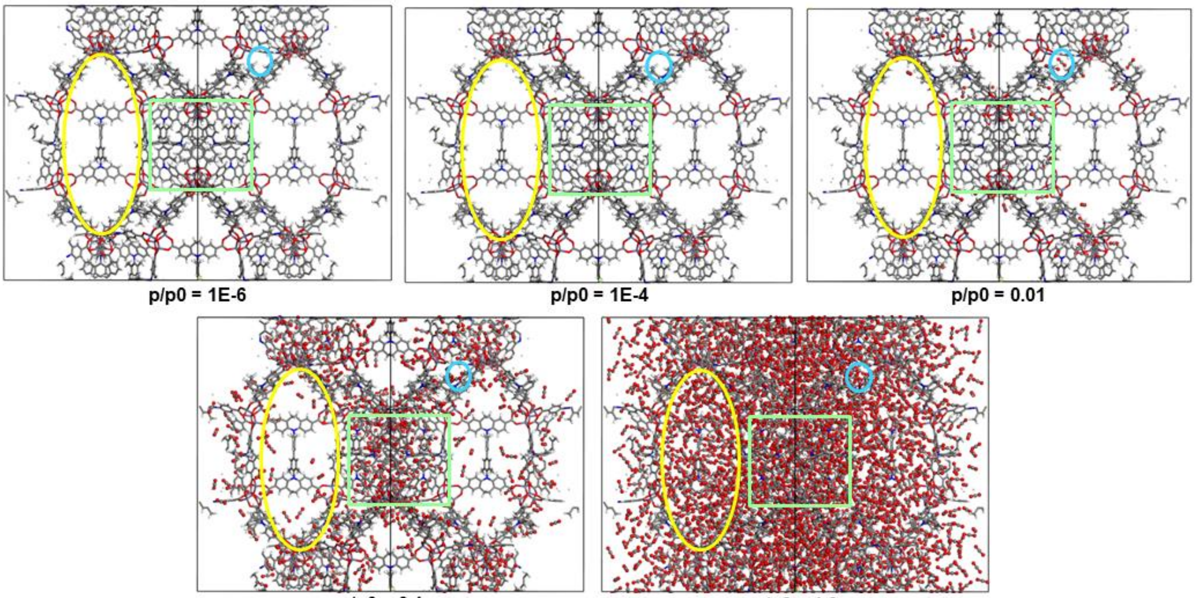

ESI Fig. S26: Illustrative GCMC configurations of CO_2 adsorption in DUT-193 at 77 K with p/p_0 ranging from 10^{-6} to 1 bar.

References:

[1] A. K. Rappe, C. J. Casewit, K. S. Colwell, W. A. Goddard, W. M. Skiff, *J. Am. Chem. Soc.* **1992**, *114*, 10024-10035.

[2] M. G. Martin, J. I. Siepmann, *J. Phys. Chem. B* **1998**, *102*, 2569-2577; J. J. Potoff, J. I. Siepmann, *AIChE J.* **2001**, *47*, 1676-1682.
